# The plastic brain: neurotoxicity of micro- and nanoplastics

**DOI:** 10.1186/s12989-020-00358-y

**Published:** 2020-06-08

**Authors:** Minne Prüst, Jonelle Meijer, Remco H. S. Westerink

**Affiliations:** Neurotoxicology Research Group, Division Toxicology, Institute for Risk Assessment Sciences (IRAS), Faculty of Veterinary Medicine, Utrecht University, NL-3508 TD Utrecht, The Netherlands

**Keywords:** Neurotoxic hazard, Plastic particles, Microplastic, Nanoplastic, Oxidative stress, Acetylcholinesterase inhibition, Nanoparticles

## Abstract

Given the global abundance and environmental persistence, exposure of humans and (aquatic) animals to micro- and nanoplastics is unavoidable. Current evidence indicates that micro- and nanoplastics can be taken up by aquatic organism as well as by mammals. Upon uptake, micro- and nanoplastics can reach the brain, although there is limited information regarding the number of particles that reaches the brain and the potential neurotoxicity of these small plastic particles.

Earlier studies indicated that metal and metal-oxide nanoparticles, such as gold (Au) and titanium dioxide (TiO_2_) nanoparticles, can also reach the brain to exert a range of neurotoxic effects. Given the similarities between these chemically inert metal(oxide) nanoparticles and plastic particles, this review aims to provide an overview of the reported neurotoxic effects of micro- and nanoplastics in different species and in vitro. The combined data, although fragmentary, indicate that exposure to micro- and nanoplastics can induce oxidative stress, potentially resulting in cellular damage and an increased vulnerability to develop neuronal disorders. Additionally, exposure to micro- and nanoplastics can result in inhibition of acetylcholinesterase activity and altered neurotransmitter levels, which both may contribute to the reported behavioral changes.

Currently, a systematic comparison of the neurotoxic effects of different particle types, shapes, sizes at different exposure concentrations and durations is lacking, but urgently needed to further elucidate the neurotoxic hazard and risk of exposure to micro- and nanoplastics.

## Background

Over the years, the environment has been contaminated with millions of tons of plastic. There are numerous different types of plastics, which are often produced for single use. The most predominant plastics are polyethylene, polypropylene, polystyrene, poly-vinyl-chloride, polyamide and polyethylene terephthalate (better known as PET). In recent years, the annual production of plastic has increased from 250 million tons in 2009 [1] to 299 million tons in 2013 [2] and 335 million tons in 2016 [1]. Approximately 10% of all annually produced plastic ends up as debris in the marine environment [3]. It is estimated that over 5 trillion pieces of plastic, ranging in size from nanoplastics to bulk plastics and weighing over 250.000 tons, are afloat at sea alone, even excluding the amount of plastic debris in fresh surface water, on terrestrial areas and on the ocean floor [4].

While plastics are very persistent, marine bulk plastics such as packaging, fishing nets and car tires are subject to fragmentation through photodegradation and erosion by wave action, contact with animals, abrasion with sand and by the water itself [4, 5]. Breakdown of these fragments contributes to the continuously increasing amount of so-called secondary microplastics (defined as particles with a diameter 0.1 μm to 5 mm) and secondary nanoplastics (defined as particles with a diameter below 100 nm). Primary micro- and nanoplastics are deliberately produced in ultra-small sizes to serve as components in cosmetics, paints, personal care products or fabrics [1, 5, 6].

Micro- and nanoplastics are found in all aquatic ecosystems [7–9], although it is still debated exactly how hazardous these materials are. Besides the potential adverse effects induced by the physical presence of micro- and nanoplastics, they can act as carrier for various (chemical) contaminants, including metals, persistent organic pollutants (POP), antibiotics and (pathogenic) micro-organisms [10–14].

In the aquatic food chain, bioaccumulation of micro- and nanoplastics occurs after ingestions by aquatic organisms, including fish and marine mammals, and subsequent transfer of engulfed particles through trophic levels [15, 16]. For nanoparticles, uptake via the gills provides an additional exposure route [17, 18]. Micro- and nanoplastics can transfer from the digestive tract and/or gills into the circulatory system [1, 18], although the exact translocation mechanisms (e.g. paracellular translocation through the tight junctions of the gut wall epithelium or transcellular via endocytosis, phagocytosis or micropinocytosis) are yet unclear. The presence of small plastic particles has subsequently been observed in organs and tissues of zooplankton [19], mussels [20], crustaceans [21] and fish [17], including the brain of fish [22], although the reported uptake is usually at low levels in aquatic species (30–50 particles in zooplankton and mussels, respectively [19, 20]).

Humans are exposed to micro- and nanoplastics via consumption of contaminated (marine) animals and other food and consumer products such as toothpaste, beer, honey, salt and sugar [14, 23–25]. Additional human oral exposure results from drinking water and from mineral water bottled in plastics and cartons [26, 27]. Additional inhalation exposure results from micro- and nanoplastics released from textiles, synthetic rubber tires and plastic covers [14, 18, 24, 25, 28].

Uptake of micro- and nanoplastics (≤0.3 μm) and subsequent translocation to the liver, spleen and lymphatic systems of rodents has been reported decades ago [29], albeit at low levels [30]. Similarly, in humans, micro-sized plastic fibers have been detected in lung tissue, indicating possible translocation of micro- and nanoplastics into the human body via particle inhalation [31]. Additionally, limited gastrointestinal uptake of biodegradable polymeric microparticles has been reported [32]. Although these combined studies highlight the possibility of uptake and translocation of micro- and nanoplastics into the human body following oral and inhalation exposure [33], there is an overall scarcity of studies that conscientiously and systematically investigated the extent of particle translocation to different organs in relation to particle dose and particle size. Moreover, the potential health risks resulting from micro- and nanoplastics exposure, uptake and translocation is poorly investigated and is an important matter of ongoing debate [14, 18, 34–36].

### Main text

#### Neurotoxicity of chemically inert metal(oxide) nanoparticles

In contrast to micro- and nanoplastics, metal(oxide) nanoparticles have been relatively well studied. Metal(oxide) nanoparticles are extensively used in food production, cosmetics, personal care products and as biomedical therapeutic agents for drug delivery and gene therapy [37–39].

Earlier studies identified the (central) nervous system as an important target for the toxic effects of metal and metal-oxide nanoparticles [39–41]. Metal and metal-oxide nanoparticles can enter the brain by crossing the blood-brain-barrier (BBB) or can surpass this barrier via retrograde transport through olfactory nerve endings [38, 42–44].

The numerous types of metal and metal-oxide nanoparticles differ in their physicochemical properties, and some of these compounds resemble the characteristics of plastic particles. Some metal nanoparticles are quite reactive and know for the ability to induce oxidative stress and subsequent damage, like iron oxide [45, 46], silver [47, 48] or copper oxide [49, 50]. However, gold (Au) and titanium dioxide (TiO_2_) nanoparticles approach the definition of chemically inert, which is an important characteristic for comparison of metal and metal-oxide nanoparticles to plastic micro- and nanoparticles [23, 28, 51].

Au-nanoparticles have been shown to translocate to brain tissue of zebrafish and adult rats, where they can induce oxidative stress, alterations of energy/mitochondrial metabolism and acetylcholinesterase (AChE) activity, and neurobehavioral effects [52–54]. Similarly, the more extensively investigated TiO_2_-nanoparticles also translocate to the brains of fish, where they can induce alterations in oxidative damage and cell death, neurotransmitter levels, locomotor behavior and spatial recognition memory [55–58]. In rodents, oral, intranasal or intratracheal exposure to TiO_2_-nanoparticles (size range 5–100 nm) resulted in oxidative stress and neuroinflammation [59, 60], dysregulation of the glutamatergic signaling and alterations in neurotransmitter levels [59, 61, 62], changes in AChE activity [60, 61], impaired motor function [63], reduction of long-term potentiation, and impairment of learning and memory [61, 64]. Additional in vitro studies confirmed the ability of TiO_2_-nanoparticles to induce oxidative stress and neuroinflammation [65–68]. See supplementary Tables 1 and 2 for details on exposure route and dose, particle size and type, and for an overview of additional studies on the neurotoxicity of Au- and TiO_2_-nanoparticles.

While some of the effects of Au-and TiO_2_-nanoparticles were observed only following exposure to high levels and/or artificial exposure route (e.g. injection), Au-and TiO2-nanoparticles can reach the brain and exert a wide range of neurotoxic effects. The extent to which these effects are also applicable to micro- and nanoplastics is however largely unknown. Given the abundance of micro- and nanoplastics and the clear neurotoxic effects of similarly sized, chemically inert Au-and TiO_2_-nanoparticles, this review examines the neurotoxic potential of micro- and nanoplastics.

### Literature search

To review the neurotoxic potential of plastic micro- and nanoparticles, a literature search was conducted to cover articles in Pubmed up to December 1, 2019 using the following combinations of search words: Neurotox* AND Microplastic* (17 hits); Neurotox* AND Nanoplastic* (4 hits); Neurotox* AND plastic particles (15 hits). In total twenty-eight papers were selected. Three additional papers were found via the bibliography of other papers. A paper was not included when the research concerned a review or when the experimental design did not include neurotoxic endpoints.

Fish and Mollusca were the most frequently researched organism groups, seventeen and six times respectively. Two studies performed research on crustaceans, two on nematodes and two on rodents, while three studies used in vitro cell cultures with various mouse and human derived neuronal cells. Sixteen studies used polystyrene plastic particles, nine studies used polyethylene particles, two studies used both polystyrene and polyethylene particles, whereas four studies used microplastics of undefined polymeric substance. Of these thirty-one studies, three studies used modified plastic particles (amino-modified, carboxylated, PEGylated). Fourteen studies investigated the (neuro) toxicity of micro or nanoplastics co-exposed with other substances; pyrene (3 times), mercury (2 times), 17-α-ethinylestradiol (EE2), Bisphenol-A (BPA), cadmium, carbamazepine, Cefalexin, chromium, Florfenicol, gold nanoparticles, Roxithromycin (ROX).

### Neurotoxic effects of micro- and nanoplastics in (marine) invertebrates

Several studies investigated the (neurotoxic) effects of polystyrene and polyethylene micro- and nanoplastics in invertebrates such as nematodes, bivalves and crustaceans, either in the presence or absence of co-exposure to other compounds. Exposure of the nematode *Caenorhabditis elegans* to five different sizes of spherical polystyrene microplastics (0.1 to 5 μm) via the culture medium (1 mg/L) resulted in excitatory toxicity on locomotor behavior, reduced survival rate and reduced average lifespan, particularly following exposure to 1.0 μm polystyrene particles. Furthermore, expression of various neuronal genes was down-regulated, which coincided with impairment of cholinergic and GABAergic neurons and oxidative stress. Unfortunately, there was no proof provided of actual uptake of the spherical polystyrene microplastics by *C. elegans* [69].

Exposure of earthworms (*Eisenia fetida*) for up to 28 days to artificial soil with low-density polyethylene particles (100–200 μm; 0.1–1.5 g/kg soil) resulted in skin damage following exposure to 1.5 g/kg soil. Particle ingestion (following 14–28 days exposure to 1.5 g/kg soil) was confirmed by extraction and counting of polyethylene particles, although the distribution of the particles within the earthworms is unknown. Exposure for 28 days to polyethylene particles at 1.0 g/kg soil, but not at 1.5 g/kg soil, resulted in increased catalase activity and malondialdehyde levels, suggesting the animals showed signs of oxidative stress. Additionally, exposure to 1.0 g/kg and 1.5 g/kg soil for respectively 21 and 28 days, increased AChE activity [70].

Exposure of freshwater zebra mussel (*Dreissena polymorpha*) to a mixture of two different sizes (1 μm and 10 μm) of virgin polystyrene microbeads at 1 and 4 × 10^6^ MPs/L for 6 days resulted in concentration of the particles in the gut lumen and subsequent transfer into tissues and hemolymph, as shown using confocal microscopy. The polystyrene microbead mixtures did not induce genotoxicity. Although both mixtures increased dopamine levels, exposure did not change the levels of serotonin and glutamate or the activity of monoamine oxidase and AChE. The low dose mixture was able to increase catalase activity and to decrease glutathione peroxidase, suggestive for (modest) cellular stress [71].

In bivalves of the species *Scrobicularia plana*, exposure to polystyrene microplastics (20 μm, 1 mg/L) resulted in the presence of particles in the hemolymph, the gills and digestive gland, as detected by optical microscopy and by infrared spectroscopy. In the gills, from 7 days exposure onwards, polystyrene microplastics induced a consistent increase in superoxide dismutase (SOD) activity as well as an increase in glutathione-S-transferase (GST) activity at the end of the exposure period, suggestive of oxidative stress. At 3–14 days of exposure as well as following depuration, AChE and lipid peroxidation (LPO) activity in the gills was also decreased. In the digestive gland from day 14 onwards, SOD activity was increased, whereas catalase activity decreased [72].

Exposure of Mediterranean mussel (*Myetilus galloprovincialis*) to polystyrene microplastics (0.11 μm, 0.005–50 mg/L) for 96 h resulted in significant alterations in expression of genes associated with biotransformation, cell-stress-response and innate immunity in the gills (*hsp70*, at 50 mg/L) and digestive gland (*cyp11*, at 0.5 mg/L; *cyp32*, at 5 mg/L; *cat*, at 0.05 and 0.5 mg/L; *lys*, at 5 mg/L). While these changes do not show a clear dose-dependence, mean value of DNA damage score was increased following exposure to 0.05-50 mg/L. Cholinesterase activity in hemolymph was decreased at 0.05–0.5 mg/L, but no other signs of neurotoxicity were observed. Unfortunately, there was no proof provided of actual uptake of the polystyrene microplastics [73].

Exposure of Mediterranean mussels (*Myetilus galloprovincialis*) to virgin and pyrene-contaminated polyethylene and polystyrene microplastics (100 μm, 1.5 g/L) for 7 days resulted in the presence of plastic particles in hemolymph, gills and gut, as detected by polarized light microscopy. Polyethylene and polystyrene microplastics induced nuclear alterations and DNA damage in addition to a reduction of AChE activity in the gills, but not the hemolymph, of the clams. The inhibition of AChE activity was not exacerbated by contamination with pyrene [74].

Exposure of Asian freshwater clams (*Corbicula fluminea*) for 96 h to Red Fluorescent Polymer Microspheres (undisclosed composition; 1–5 μm, 0.2 or 0.7 mg/L) resulted in the presence of plastic particles in the gut, digestive gland lumen, connective tissue, hemolymphatic sinuses, and gills surface as detected by both light and fluorescence microscopy. Exposure to 0.2 but not to 0.7 mg/L polymer microspheres significantly inhibited cholinesterase activity, which was further exacerbated by co-exposure to florfenicol [75]. In a comparable study, Asian freshwater clams were exposed for 8 days to Red Fluorescent Polymer Microspheres (1–5 μm, 0.13 mg/L), resulting in particle presence in the digestive tract and gills. Exposure to the polymer microspheres reduced cholinesterase activity and increased LPO levels suggestive for oxidative damage. These effects were only partly reversible following six days of recovery. Surprisingly, the observed effects were alleviated by co-exposure to mercury [76].

Exposure of larvae of the Striped barnacle (*Amphibalanu ampitrite*) and Brine shrimp (*Artemia fransiscana)* to 0.1 μm fluorescent polystyrene microparticles (0.001–10 mg/L) for 24 and 48 h resulted in the presence of plastic particles in the gut, as detected by fluorescence microscopy. It is however unclear if the particles were able to reach the surrounding tissues. Microplastic exposure (≥1 mg/L) for 48 h induced alterations in the swimming speed. Moreover, microplastic exposure resulted in miscellaneous effects on enzyme activity. Catalase activity was mainly increased, in particular at the high dose (1 mg/L), whereas effects on cholinesterase (acetylcholinesterase and propionylcholinesterase) appeared more random without a clear dose-dependency [77].

Exposure of larvae of the Brine shrimp (*Artemia fransiscana)* to amino-modified polystyrene nanoparticles (50 nm, 0.1–10 μg/mL) for 48 h or 14 days resulted in decreased GST and catalase activity, suggestive for oxidative stress, as well as carboxylesterase and ChE inhibition at 1 μg/mL. Unfortunately, there was no proof of actual uptake of the polystyrene nanoplastics [78].

### Neurotoxic effects of micro- and nanoplastics in fish

Several articles reported on the neurotoxicity of polystyrene and polyethylene micro- and nanoplastics in fish in the absence of co-exposure to other compounds. Exposure of adult Japanese rice fish (*Oryzias latipes*) to fluorescent polystyrene nanoplastics (40 nm, 10 mg/L) for 7 days showed the presence of particles primarily in the gills and intestine but also in testis, liver, and blood, as detected by fluorescence microscopy. Notably, particles were also detected in the brain, indicating that nanoplastics have the innate capacity to cross the BBB. While particle concentrations were determined in blood, amounting to 16.5 and 10.5 ng/mg blood protein for respectively males and females, particle concentrations have unfortunately not been determined in brain (or other tissues) [79].

Furthermore, presence of fluorescent polystyrene particles (0.1 μm, 1–100 μg/L) was observed using fluorescence spectrophotometry in lyophilized gut, gills, liver and brain tissue of adult tilapia fish (*Oreochromis niloticus*) following 1–14 days of exposure. The presence of polystyrene microparticles was paralleled by an inhibition of AChE activity as well as by induction of SOD [80].

Exposure of Crucian carp (*Carassius carassius*) to amino-modified polystyrene micro- and nanoparticles (53 nm and 180 nm, 100 mg/L), via trophic transfer in the aquatic food chain for 64 days, resulted in the presence of polystyrene particles in the fish brain as detected using hyperspectral microscopy. Nanoparticles had a higher presence in the brain than the microparticles. The presence of polystyrene micro- and nanoparticles in the brain coincided with alterations in behavioral patterns, decreased brain mass and morphological changes in the cerebral gyri, which was most profound for the nanoparticles [22].

In contrast, juvenile surgeonfish (*Acanthurus triostegus*) exposed to polystyrene microplastics (90 μm, 5 × 10^3^ particles/L (~ 0.81 mg/L)) for up to 8 days did not show alterations in (foraging) behavior, body weight or susceptibility to predation, although ingestion was confirmed using microscopy analysis [81]. However, animal species, exposure duration, particle number and particle size as are all very different between these studies, so it remains to be elucidated which factor(s) determine the neurotoxic hazard.

Comparable contradictions are observed following exposure to polyethylene microplastics. Following exposure of zebrafish larvae (*Danio rerio*) to low-density polyethylene microplastics (10 μm, 5–500 μg/L, for 10–20 days), particle presence was observed using bright-field microscopy only in the intestine, but not in the brain. The exposure however had minimal effects on growth or gene expression, including the gene for AChE [82]. Another study using adult zebrafish showed that exposure to high-density, fluorescent polyethylene microplastics (size range 10–22 μm up to 500–600 μm; 11–1100 particles/L) resulted in particle ingestion and presence in gills and intestine as shown using fluorescence microscopy. Importantly, exposure (≥110 particles/L) resulted in changes in locomotory behavior and even induced seizures (1100 particles/L) [83].

Exposure of discus fish (*Symphysodon aequifasciatus)* for up to 30 days to fluorescent polyethylene microplastics (70–88 μm, 200 μg/L) also showed particle presence in the body, although the exact location is unknown as particle detection was performed using fluorescence spectrometer recordings following lyophilization of the exposed fish. Particle exposure coincided with reduced activity of AChE and changes in some digestive enzymes. Notably, the particle concentration was higher in fish exposed at 31 °C compared to fish exposed at 28 °C, although the difference in exposure temperature did not affect AChE inhibition [84].

Other studies investigated the effects of exposure to micro- and nanoplastics in the presence of other (environmental) toxicants. Exposure of zebrafish larvae to polystyrene nano- and microplastics (47 nm and 41 μm, 1 mg/L) for 5 days, in the presence or absence of 17-α-ethynylestradiol (2 and 20 μg/L), resulted in particle presence in the body, although the exact location is unknown as particle detection was performed using fluorescence recordings following lyophilization of the exposed fish. Fish co-exposed to a high concentration of 17-α-ethynylestradiol showed less particle presence. Exposure to microplastics alone did not evoke major effects, although exposure to nanoplastics alone reduced body length and inhibited locomotion and AChE activity. Co-exposure to 17-α-ethynylestradiol did not exacerbate the effects of micro- and nanoplastics, rather plastics alleviated the effects of 17-α-ethynylestradiol, likely by decreasing the concentration of freely dissolved 17-α-ethynylestradiol [85].

In adult zebrafish exposed to fluorescent polystyrene nanoplastics (50 nm, 1 mg/L) for 1–3 days, plastic particles were observed in the brain, gills and muscle using fluorescence spectrometer measurements of lyophilized tissues. Co-exposure to polystyrene nanoplastics with Bisphenol A (BPA, 1 μg/L) increased the concentration of BPA in the brain compared to BPA alone, confirming that micro- and nanoplastics can act as carrier for BPA. Exposure to BPA alone or polystyrene nanoparticles alone led to inhibition of AChE, whereas the inhibition of AChE was attenuated following co-exposure to nanoparticles with BPA. In contrast, Bisphenol A (BPA, 1 μg/L) alone or polystyrene nanoparticles alone did not affect dopamine levels, whereas co-exposure resulted in a ~ 2-fold increase in dopamine levels on the first day of exposure only [86].

In juvenile barramundi fish, no changes in movement and predatory behavior were observed after exposure to polystyrene microplastics (97 μm, 100 particles/L). Unfortunately, there was no proof of actual uptake of the polystyrene nanoplastics. Combined exposure of microplastics with pyrene (100 nM) resulted in decreased swimming movement, although this effect was minor compared to the effect of pyrene alone [87].

Exposure of red tilapia to roxithromycin (ROX, 50 μg/L) alone resulted in decreased AChE, which was attenuated in combination with fluorescent polystyrene microplastics (0.1 μm, 1–100 μg/L), suggesting a decrease in neurotoxicity or more likely in the bioavailability of ROX in the presence of polystyrene microplastics. The presence of microplastics was confirmed in the gut, gills and to a lesser extent also brain and liver using fluorescence microscopy. However, the study unfortunately did not include an group exposed to microplastics in the absence of ROX, making it difficult to draw any conclusions regarding the neurotoxicity of microplastics [88].

Exposure of Sea bass to microplastics (1–5 μm, 0.69 mg/L) resulted in inhibition of AChE activity in the brain, but not of ChE in muscle. Additionally, an increase in LPO was found in the brain and muscle of the fish following exposure to microplastics alone. Both effects were exacerbated by co-exposure to mercury (10 and 16 μg/L), although there was no clear dose-dependence. Unfortunately, there was no proof provided of actual uptake of the microplastics [16].

In juveniles of the common goby (*Pomatochistus microps*) exposure to fluorescent polyethylene microplastics (1–5 μm, 0.216 mg/L) also resulted in inhibition of AChE activity, which was exacerbated by co-exposure to chromium (5.6–28.4 mg/L) [89]. Contrary, co-exposure of polyethylene microplastics (1–5 μm, 0.18 mg/L MPs) with cadmium (3–50 mg/L, [90]) or pyrene (200 μg/L, [91]) did not exacerbate AChE inhibition. Unfortunately, there was no proof provided of actual uptake of the polyethylene microplastics [89–91].

The inhibition of AChE by fluorescent polyethylene microplastics (1–5 μm, 0.18 mg/L MPs) may be temperature-dependent, at least in some species, as exposure to microplastics alone at 20 °C did not alter AChE activity, whereas exposure at 25 °C induced a mild increase in LPO and inhibition of AChE activity [92]. A similar temperature-dependence was observed for effects of fluorescent polyethylene microplastics (1–5 μm, 0.18 mg/L MPs) on predatory behavior, LPO and AChE inhibition. At 25 °C, exposure to microplastics alone decreased predatory behavior and AChE inhibition and increased mortality and LPO significantly compared to fish living in water of 20 °C [93]. Unfortunately, there was no proof provided of actual uptake of the polyethylene microplastics [92, 93].

Most of these studies that used co-exposures used only one dose of nano- or microplastic and/or one dose of chemical, making it difficult to assess the contribution of the plastic particles to the observed effect(s). Additionally, the presence of plastic particles likely alters the free concentration of the co-exposed chemical, further hampering interpretation of the results.

See Table 1 for details.

**Table 1:** Overview of the literature investigating neurotoxic effects of micro- and nanoplastics. The particle concentration is only mentioned for micro- and nanoplastics or for mixtures containing micro- and nanoplastics. The reported particle size reflects the diameter of primary particles. Every study included a control group that was not exposed to micro- and nanoplastics or any other substance, or measurements were taken at timepoint 0, before exposure

Model system	Particle type / size	Exposure method	Exposure dose	(Neuro)toxic effects	Ref.
**Nematodes**					
*Caenorhabditis elegans*	PS-MPs of 0.1, 0.5, 1, 2 & 5 μm	in medium, for 3 days	1 mg/L medium	Excitatory toxicity on locomotive behaviour. Damage to cholinergic and GABAergic neurons, oxidative stress; no clear size-dependence).	[69]
Earthworm *(Eisenia fetida)*	PE-MPs of 100–200 μm	in soil, for 7–28 days	0.1, 0.25, 0.5, 1.0 and 1.5 g/kg soil	Particle ingestion at 1.5 g/kg. Skin damage (1.5 g/kg). Increased AChE activity (≥1.0 g/kg and 1.5 g/kg at respectively 21 and 28 days). Increased CAT activity and MDA levels (1.0 g/kg, 28 days).	[70]
**Bivalves**					
Zebra mussel (*Dressene polymorpha*)	PS-MPs mixture of 1 & 10 μm (1:1)	In water, for 3 & 6 days	1 × 10^6^ MPs/L (mix 1) 4 × 10^6^ MPs/L (mix 2)	Particle presence in hemolymph and tissues. Increased DA levels (mix 1, 3 days & mix 2, 6 days). Cellular stress (decreased CAT; mix 1, 6 days). No change in AChE or MAO activity or in Glu and 5-HT levels. No genotoxicity.	[71]
Peppery furrow shell (*Scrobicularia plana*)	PS-MPs of 20 μm	In water, for 14 days followed by 7 days of depuration	1 mg/L (~ 4000 particles/L)	Particle presence in hemolymph, gills and digestive gland. Increased SOD (gills, ≥7 days exposure; digestive gland, ≥14 days). AChE and LPO activity decreased (gills, 3–14 days). CAT activity decreased (digestive gland, ≥3 days).	[72]
Mediterranean mussel (*Mytilus galloprovincialis*)	PS-MPs of 0.11 μm	In water, for 96 h	0.005, 0.05, 0.5, 5 and 50 mg/L MPs alone, and mixture of 0.05 mg/L PS-MPs + 6.3 μg/L Cbz	Altered gene expression (MPs alone and MPs with Cbz; ≥ 0.05 mg/L). ChE inhibition in hemolymph (MPs 0.05 and 0.5 mg/L). DNA damage (≥ 0.05 mg/L).	[73]
Mediterranean mussel (*Mytilus galloprovincialis*)	PE-MPs and PS-MPs of 100 μm	In water, for 7 days	1.5 g/L (with and without 50 μg/L pyrene)	Particle presence in hemolymph, gills and gut. Reduction of AChE activity in gills (PE and PS). Nuclear alterations and DNA damage, but no changes in oxidative stress markers (GST, CAT, LPO).	[74]
Asian clam (*Corbicula flumenia)*	MPs (Red Fluorescent Polymer Microspheres)*of 1–5 μm	In water, for 96 h	0.2 mg/L (~ 37.000 particles/L) and 0.7 mg/L (~ 128.500 particles), with or without 1.8 mg/L and 7.1 mg/L Florfenicol (antimicrobial agent)	Particle presence in gut, digestive gland lumen, connective tissue, hemolymphatic sinuses, and gills surface. Inhibition (31%) of ChE activity (0.2 mg/L, but not at 0.7 mg/L), which was exacerbated by Florfenicol.	[75]
Asian clam (*Corbicula flumenia)*	MPs (Red Fluorescent Polymer Microspheres)*of 1–5 μm	In water, for 8 days	0.13 mg/L (~ 24.000 particles/L), with or without 30 μg/L mercury	Particle presence in digestive tract and gills. Inhibition (15%) of ChE activity. Increased (~ 2-fold) LPO levels suggestive for oxidative stress (LPO).	[76]
**Crustaceans**					
Striped barnacle (*Amphibalanu ampitrite)*	PS-MPs (fluorescent) of 0.1 μm	In water, for 24 and 48 h	0.001, 0.01, 0.1, 1, 10 mg/L	Particle presence in gut. Decreased swimming speed (≥ 1 mg/L). Increase in ChE activity (0.001–0.1 mg/L). Decrease in (P)ChE activity (1 mg/L). Increase in CAT activity (0.1–1.0 mg/L).	[77]
Brine shrimp (*Artemia fransiscana)*	PS-MPs (fluorescent) of 0.1 μm	In water, for 24 and 48 h	0.001, 0.01, 0.1, 1, 10 mg/L	Particle presence in gut. Increased swimming speed (≥ 1 mg/L). Decrease in AChE activity (0.001–0.01 mg/L). Increase in PChE activity (0.01–0.1 mg/L). Increase in CAT activity (0.001–1.0 mg/L).	[77]
Brine shrimp (*Artemia fransiscana)*	PS-NH_2_ NPs of 50 nm	In water, for 48 h up to 14 days	0.1, 1.0, 3.0 and 10.0 mg/L	ChE activity decreased (1 mg/L). CbE activity decreased (1 mg/L). GST decreased (1 mg/L). CAT decreased (1 mg/L).	[78]
**Fish**					
Japanese rice fish (*Oryzias latipes*)	PS-NPs of 40 nm	In medium, for 7 days	10 mg/L	Particle presence in gills, intestine, testis, liver, blood and brain, suggesting penetration of BBB.	[79]
Red tilapia (*Oreochromis niloticus*)	PS-MPs of 0.1 μm	In medium, for 1–14 days	1, 10 and 100 μg/L	Particle presence in gut, gills, liver and brain tissue (≥ 1 μg/L, ≥ 6 days). Inhibition of AChE activity (37.7%) in brain (≥ 1 μg/L, ≥ 3 days). Antioxidant enzyme induction (SOD; ≥ 1 μg/L, 1 days > 3–14 days); no change in MDA levels.	[80]
Crucian carp (*Carassius carassius*)	positively charged amino-modified PS-NP and -MP of 53 and 180 nm	In water, for 64 days or via PS-NP fed crustaceans	100 mg/L	Particle presence in brain (53 nm and 180 nm). brain weight loss (53 nm and 180 nm). Behavioural changes and enlarged cerebral gyri (53 nm).	[22]
Convict surgeonfish, juvenile (*Acanthurus triostegus*)	PS-MPs of 90 μm	In water, for 8 days	0.81 mg/L (~ 5000 particles/L)	Particle presence in digestive tract. No effect on foraging behaviour, body weight or survival rate when exposed to a predator.	[81]
Zebrafish, juvenile (*Danio rerio)*	PE-MPs of 10 μm	In water, for 10 and 20 days	5, 50 and 500 μg/L (or 1040, 10,400 and 104,000 particles/L)	Particle presence in intestine, but not in brain or other organs. No changes in growth or *ache* gene expression.	[82]
Zebrafish, adult (*Danio rerio)*	PE-MPs of 10–22, 45–53, 90–106, 212–250 and 500–600 μm	In water, for 96 h	11, 110, 1.100 MPs/L	Ingestion and particle presence in intestine and gills (19.7–558.4 μm). Altered locomotive behaviour (≥ 110 MPs/L) and induction of seizures (≥1100 MPs/L). No changes in mortality.	[83]
Discus fish (*Symphysodon aequifasciatus)*	PE-MPs of 70–88 μm	In water, for 30 days (28 °C and 31 °C)	200 μg/L	Particle presence in body (31 °C > 28 °C). Decreased AChE in head (both 28 °C and 31 °C). No changes in growth or survival rate.	[84]
Zebrafish, larvae (*Danio rerio*)	PS-NPs of 47 nm, PS-MPs of 41 μm	In water, for 5 days	1 mg/L, with or without 2 and 20 μg/L EE2	Particle presence in body. Inhibition of AChE by 9% (MPs), 40% (NPs) 21% (MP and NP co-exposed with EE2); locomotor hypoactivity 22% (NPs) and 18–36% (co-exposed with EE2).	[85]
Zebrafish, larvae (*Danio rerio*)	PS-NPs of 50 nm	In water, for 3 days	1 mg/L, with or without 0.78 and 1.0 μg/L BPA	Particle presence in head, gills and muscle. Decreased AChE activity 46% (NPs alone) and increased DA levels (only for mixture of PS-NP with BPA).	[86]
Barramundi, juvenile (*Lates calcarifer)*	PS-MPs of 97 μm	In water, for 24 h	100 MPs/L, with or without 100 nM Pyrene	Little (co-exposure) or no (PS-MPs alone) effect on swimming movement or foraging behaviour.	[87]
Red tilapia (*Oreochromis niloticus*)	PS-MPs of 0.1 μm	In water, for 1–14 days	1, 10 and 100 μg/L, with 50 μg/L ROX	Particle presence in gut, gills and to a lesser extent also brain and liver. Decrease in AChE activity (co-exposed, ≥ 1 μg/L). Note: there was no ‘MP only’ group.	[88]
Sea bass, juvenile (*Dicentrarchus labrax*	MPs* of 1–5 μm	In water, for 96 h	0.26 and 0.69 mg/L, with or without 10 and 16 μg/L mercury	Inhibition of AChE activity (50%) and increased LPO in the brain (0.69 mg/L MPs). Inhibition of AChE (64–76%) and increased LPO in brain exacerbated by co-exposure (mercury, all concentrations).	[16]
Common goby, juvenile (*Pomatoschistus microps*)	PE-MPs of 1–5 μm	In water, for 96 h	0.216 mg/L, with or without 5.6–28.4 mg/L chromium	AChE activity decreased with 20% (MPs alone) and 31% (co-exposed with chromium).	[89]
Common goby, juvenile (*Pomatoschistus microps)*	PE-MPs of 1–5 μm	In water, for 96 h	0.18 mg/L, with or without 3–50 mg/L cadmium	Increased mortality (MP alone and in mixture with Cd); decreased AChE activity (MP alone and mixture MP with 3, 6 and 13 mg/L); behavioural inhibition (MP alone and mixture MP with 3, 6 and 13 mg/L Cd); no oxidative stress (LPO and GST).	[90]
Common goby, juveline (*Pomatoschistus microps)*	PE-MPs of 1–5 μm	In water, for 96 h	18.4 μg/L and 0.18 mg/L, with or without 200 μg/L pyrene	Decrease in AChE activity (22%) (MP alone and co-exposed) (18.4 μg/L = 184 μg/L).	[91]
Common goby, juvenile (*Pomatoschistus microps*	PE-MPs of 1–5 μm	In water, for 96 h	0.18 mg/L, with or without 0.2 mg/L Au0-NP	Insignificant AChE activity inhibition (13%); oxidative stress (LPO, GST) (25 °C, not 20 °C).	[92]
Common goby, juveline (*Pomatoschistus microps)*	PE-MPs of 1–5 μm	In water, for 96 h	0.18 mg/L, with or without 1.3–10 mg/L cefalexin	Decrease in AChE (8% at 20 °C, 21% at 25 °C), behavioural inhibition (28% at 25 °C) and mortality (33% 25 °C); mixture increased toxicity of MPs and cefalexin.	[93]
**Mammals**					
Mouse (*Mus musculus*)	PS-MPs of 5 μm and 20 μm	Oral gavage for 30 days	0.01–0.5 mg/day (~ 0.5–25 mg/kg body weight/day, assuming bodyweight of 20 g). (1 × 10^5^–5 × 10^6^ 5 μm particles / 2 × 10^3^–1 × 10^5^ 20 μm particles)	Particle presence in gut, liver and kidney. In liver, dose-dependent increase in AChE, LDH, GSH-Px and SOD activity; dose-dependent decrease in ATP and CAT in liver (≥ 0.01 mg/day, both 5 and 20 μm).	[94]
Wistar rat, male (*Rattus norvegicus domestica*)	PS-MPs of 40 nm	Oral gavage for 35 days	1, 3, 6 and 10 mg/kg body weight/day	No alterations in behaviour or body weight gain.	[95]
**Cell cultures**					
Human-derived cerebral cell line (T98G) and epithelial cells (HeLa)	PE-MPs of 3–16 μm, PS-MPs of 10 μm	In culture medium, for 24 h	0.05, 0.1, 1, 10 mg/L	ROS generation (PS only at 10 mg/L; both cell lines). No changes in cell viability.	[96]
Primary mouse astrocytes, neurons, microglia and brain vascular endothelial cells	PS-PEG and PS-COOH NPs of 55 nm	In culture medium, for 24 h	7.8–250 mg/L (or 3 × 10^13^ up to 1 × 10^15^ NPs/L)	Decreased mitochondrial activity and cell viability (≥ 250 mg/L). Internalization of NPs (2 × 10^14^ NPs/L).	[97]
Human-derived embryonic stem cell (3D model)	PE-NPs of 33 nm	In culture medium, for 84 h and for 18 days	22.5, 45, 90, 180, 360, 720 and 1440 mg/L (48 h), 22.5, 45, 90, 180, 360 mg/L (18 days)	48-h exposure: Penetration of NPs into 3D structure, internalization of NPs (≥ 360 mg/L). Increased cytotoxicity and oxidative stress (dose-dependent). 18-day exposure: PE-NP accumulation (≥ 22.6 mg/L). Altered gene expression (22.5 mg/L) and increased cytotoxicity (≥ 180 μg/mL).	[98]

*Abbreviations*: *5-HT* serotonin, *AChE* acetylcholinesterase, *Au* gold, *BBB* blood-brain barrier, *BPA* Bisphenol-A, *CAT* catalase, *CbE* carboxylesterase, *Cbz* carbamazepine, *Cd* cadmium, cholinesterase, *DA* dopamine, *EE2* 17α-ethinylestradiol, *Glu* glutamate, *GST* glutathione-S-transferase, *LPO* lipid peroxidation, *MAO* monoamine oxidase, *MDA* malondialdehyde, *MP* microplastics, *NP* nanoplastics, *PChE* propionylcholinesterase, *PE* polyethylene, *PS* polystyrene, *ROX* Roxithromycin, *SOD* superoxide dismutase. Asterisks (*) indicate the composition of the plastic particles is not disclosed

### Neurotoxic effects of micro- and nanoplastics in rodents

In striking contrast to the relative wealth of available rodent in vivo studies with metal(oxide) nanoparticles, there are only two studies that investigated the neurotoxicity of micro- and nanoplastics in rodents. This is particularly striking given the observed neurotoxic effects of exposure to micro- and nanoplastics in fish and (marine) invertebrates. In the only published in vivo mice study, adult mice were chronically (30 days) exposed to polystyrene microplastics (5 and 20 μm, 0.01–0.5 mg/day (~ 0.5–25 mg/kg body weight/day)) via oral gavage. Exposure to polystyrene microplastics resulted in uptake and particle presence in the gut, liver and kidneys of the mice, as determined using fluorescence spectrometer measurements of lyophilized tissues. Particle concentrations in tissues increased rapidly during the first week of exposure and plateaued at ~ 0.2, 1.0 and 1.4 mg/g for 5 μm particles in liver, kidney and gut respectively. Particle concentration was much more uniform for 20 μm particles, which plateaued at ~ 0.8 for liver, kidney and gut. Examination of the liver indicated dose-dependent changes in energy metabolism (decreased ATP levels, increased LDH activity) and oxidative stress (increased GSH-Px and SOD, decreased CAT). Interestingly, AChE activity in liver increased, whereas metabolomic alterations also suggested potential changes in neurotransmitter levels. Notably, there was limited difference in effect size between 5 μm and 20 μm particles (mass-based). Unfortunately, brain tissue was not investigated [94].

The other in vivo study involved chronic (5 weeks) exposure of male rats to high doses of polystyrene nanoplastics (40 nm, 1–10 mg/kg body weight/day). However, exposure did not result in behavioral alterations or changes in body weight gain. Unfortunately, there was no proof provided of actual uptake of the polystyrene nanoplastics [95].

See Table 1 for details.

### Neurotoxic effects of micro- and nanoplastics in vitro

Comparable to rodent in vivo studies, there is a striking scarcity of mechanistic in vitro studies into the potential neurotoxic effects of exposure to micro- and nanoplastics. To date, only three studies investigated the neurotoxicity of micro- and nanoplastics exposure in vitro. See Table 1 for details.

Human T98G cerebral cells and human epithelial HeLa cells both show increased production of reactive oxygen species (ROS) upon exposure (24 h) to polystyrene microplastics (10 μm, 0.05–10 mg/L), but only at the highest concentration tested. Exposure to polyethylene microplastics (3–16 μm, 0.05–10 mg/L) did not result in increased ROS production [96].

An earlier study investigated the effects of exposure of five murine neuronal cell types to core-labeled polystyrene nanoplastics (55 nm, 7.8–250 mg/L) and showed that nanoplastics can affect mitochondrial activity and LDH leakage of neuronal cells, although only at very high concentrations (250 mg/L). This study also indicated that toxicity of polystyrene nanoplastics increased for ‘aged’ particles (stored for > 6 months) compared to ‘fresh’ particles, possibly due to particle aggregation and/or adsorption of bioactive compounds. Moreover, the study revealed using confocal fluorescence microscopy that microglial cells were able to internalize carboxylated polystyrene nanoparticles by phagocytosis, suggesting the potential for neuroinflammation, as also observed following exposure to metal(oxide) nanoparticles. Interestingly, in contrast to carboxylated nanoparticles, PEGylated nanoparticles were hardly internalized by microglial cells [97].

Internalization of particles has also been shown for polyethylene nanoplastics (33 nm) in human dopaminergic neurons and developing neurospheres following respectively semi-acute (48 h, 22.5–1440 mg/L) and chronic (18 days, 22.5–360 mg/L) exposure. Internalization of polyethylene nanoplastics, as detected using fluorescence imaging and flow cytometry, coincided with altered gene expression and increased malondialdehyde (MDA) levels, suggestive of oxidative stress. At high doses (≥180 mg/L), exposure resulted in decreased cell viability [98].

### General toxicity of micro- and nanoplastics

Besides these findings on the neurotoxicity of micro- and nanoplastics, a wide range of toxic effects in diverse species have been reported. These can be summarized as alterations in gene expression [69, 71, 74, 78, 85, 86, 92], inflammation of gut, gills, liver, kidney and/or muscle [16, 72], particle accumulation in tissues of gills, intestine, liver, kidneys, gallbladder and/or gonads [72, 79, 80, 86], (lipid) oxidative damage in body/organs [16, 72, 76–78, 85, 94], disturbed metabolism [74, 94, 99], alterations in motility and behavior [22, 69, 77, 89], alterations in intestinal barrier function and gut microbiome [99], reduction of overall fitness [76] and increased mortality [78, 89]. For additional reviews on the additional non-neurotoxic effects of micro- and nanoplastics see [34, 100, 101].

### Factors influencing neurotoxic potential of micro- and nanoplastics

Various factors can be identified that could affect the neurotoxicity of micro- and nanoplastics. Obviously, the magnitude to which organisms are exposed to particles is an important factor influencing the potential neurotoxicity of plastic particles [102]. However, current exposure levels are much lower than those used in experimental settings. On the other hand, the exposure duration in experimental settings is often much shorter than relevant for realistic (human) exposure, even though some studies indicate that the neurotoxic effects of micro- and nanoplastics depend on the exposure duration [78, 80, 88]. Next to exposure concentration and duration, exposure temperature may also affect the neurotoxicity of micro- and nanoplastics, at least in fish, with increased toxicity at higher temperatures [92, 93].

In addition to the abovementioned exposure characteristics, neurotoxicity of micro- and nanoplastics may also be strongly affected by particle characteristics. Particle size is assumed to be among the most crucial characteristics. Generally, nanoparticles are more easily taken up and have a higher toxic potential than microparticles [20, 102]. With respect to plastic particles, there is only fragmentary data to support the notion that smaller sized particles exert more toxicity (e.g., [22, 94], but also see [69].

Also the hydrodynamic diameter of particles, or secondary particle size, may be of importance with respect to particle neurotoxicity. While smaller particles may seem more neurotoxic, they are also more likely to cluster together and form aggregates. While aggregation increases (secondary) particle size and would theoretically lower the neurotoxic potential, this is hardly studied. One study even showed that six months aged nanoplastics increased size from 65 nm to more than 1300 nm and induced more toxic effects than the original particles, suggesting that aggregated particles may actually have a higher neurotoxic potential [97].

The degree of aggregation depends on particle surface charge and the suspension medium [57, 65, 66, 77, 78]. However, particle surface charge may also directly influence biological activity and neurotoxic potential of micro- and nanoplastics [28, 51]. For example for Au-nanoparticles it has been shown that a negative surface charge is associated with higher cellular internalization [103], while positive surface charge increases the disruption of plasma membranes and causes more mitochondrial damage [104]. Unfortunately, only few studies analyzed the surface charge of the micro- and nanoplastics used (with zeta-potentials ranging from + 40 mV to − 50 mV) and assessing the effect of surface charge on the neurotoxicity of micro- and nanoplastics is still in its infancy.

For metal(oxide) particles, the toxicity of the particles depends to some degree on the type of metal. Similarly, it is likely that the chemical composition of the micro- and nanoplastics will affect the neurotoxic potential. Although a direct comparison is currently lacking, the shape of the plastic particles may also be of comparable importance as the different shapes (spheres, fibers and rods) also differ considerably in for example surface area and internalization potential [51].

Confirmation of the above-mentioned notions thus requires extensive research that is focused on a direct comparison of the neurotoxic effects of different types of differently sized and shaped particles, also taking into account aggregation, using a range of in vivo and in vitro model systems. An additional complicating factor is the potential of micro- and nanoplastics to act as a vector for chemicals and pathogens. Although not fully elucidated, micro- and nanoplastics may adsorb environmental chemicals [12] and even pathogens [105]. As such, micro- and nanoplastics may facilitate exposure to these potential harmful agents, indirectly exacerbating (neuro)toxicity.

### Reflections on and potential implications of neurotoxicity induced by micro- and nanoplastics

Still only limited data on the neurotoxic effects of micro- and nanoplastics are available and often the effects of micro- and nanoplastics have been assessed with co-exposure to other (environmental) contaminants. The studies that investigated co-exposures are often difficult to interpret as often only one dose of nano- or microplastic and/or one dose of chemical is used, whereas effects of plastic particles on the free concentration of the co-exposed chemical are not assessed.

Information regarding levels of small plastic particles in the environment, (drinking) water and food chain are still scarce and often only limited quality criteria are reported. More and improved data on the occurrence of small plastics particles in the different environmental matrices is needed to reliably estimate human exposure and aid hazard and risk assessment, and current efforts aim at harmonizing monitoring methods and quality criteria [106–108].

The concentrations of micro- and nanoplastics used in experimental studies are often (much) higher than those currently found in the (aquatic) environment. In the described experiments, concentrations ranged from 1 μg/L (~ 1.8 × 10^9^ particles/L) [80] to 250 mg/L (~ 10 × 10^15^ particles/L) [97]. Unfortunately, the dose is often expressed as weight/volume, without info on particle density. Consequently, information regarding particle numbers is often unknown. Although exact details on human intake of micro- and nanoplastics are often also unknown, these are likely to be much lower. For example, annual human intake of microplastics via (shell) fish is expected to be around 11.000 particles per person for Europeans who often consume seafood, whereas the annual plastic particle intake via salt is approximated at 37 particles per individual [109]. Atmospheric exposure appears also modest with 0.4 to 60 plastic microparticles/m^3^ indoor and 0.3 to 1.5 microparticles/m^3^ outdoors [28]. Notably, while some information is available on (human) intake, the information regarding uptake and translocation in animals or human is even more scarce. Few of the studies published so far made serious efforts to quantify particle uptake and translocation, so it is often unclear whether or not the particles actually made it to the tissues/systemic circulation, whether or not particles can subsequently be excreted/eliminated, and how uptake and distribution relate to the observed (neurotoxic) effect.

Another challenge for assessing the neurotoxic hazard of plastic particles relates to the facts that most studies used manufactured, spherical polystyrene particles to assess (neuro) toxicity, while polypropylene, polyester and polyamide (irregular shaped) particles and fibers were more frequently discovered in organisms collected in the field than polystyrene [3, 9]. Also, most studies involved (pristine) microplastics (≥0.1 μm), and mostly fluorescent to ease detection, although such particles may be less relevant for neurotoxic hazard characterisation.

The detection of small plastic particles is a challenge on its own. Microplastics, especially those that are fluorescent and/or larger (1 μm), can be detected using patience and microscopy approaches, including confocal, bright-field, polarized light and/or fluorescence imaging. These approaches can provide information on particle number and location (in e.g. tissue slices), but lack throughput. For alternative approaches, such as flow cytometry and fluorescent spectrometry, organisms/tissues first need to be lyophilized. Consequently, information regarding the exact location of the particles is lost. Reliable detection and localization of nanoplastics, especially if these are not labeled, will prove even more troublesome and this is likely the challenge for the field for the coming years.

Despite these limitations and the often observed absence of a clear dose-dependence of the effects (in particular for gene expression studies), there seem to be several consistent effects. Several studies reported the accumulation of micro- and nanoplastics in brain tissue of fish and indications that micro- and nanoplastics can cross the blood-brain barrier [22, 79, 80, 86]. This is confirmed by additional literature observing blood-brain barrier permeability for polystyrene nanoparticles in vivo [110] and internalization in neuronal cells in vitro [97, 98, 111, 112]. Overall, these findings highlight that (human) exposure to micro- and nanoplastics can result in systemic uptake and/or accumulation in the brain.

An increase of LPO levels is a reliable indicator for oxidative stress. Several studies demonstrated increased LPO levels in marine invertebrates, in the brain of fish and in neuronal cells in vitro following exposure to (different types of) micro- and nanoplastics, indicating that exposure to plastic particles induces oxidative stress (Table 1). This is confirmed by the increase in ROS in vitro after 24 h [96]. Overall, these results suggest that micro- and nanoplastics, just like metal(oxide) nanoparticles (Supplementary Tables S1 and S2), have the potential to induce oxidative stress in cells of the nervous system. This is of considerable concern as uncontrolled ROS can affect various (intra) cellular processes, such as protein oxidation, nuclear DNA damage, LPO levels, cell membrane destabilization, damage to mitochondrial proteins, endoplasmic reticulum stress and subsequent cellular damage including cell death and neuroinflammation [113]. Notably, oxidative stress and inflammation in the (central) nervous system have been linked to various neurodegenerative diseases, such as Alzheimer’s Disease, Parkinson’s Disease, Huntington’s Disease and Amyotrophic Lateral Sclerosis [113, 114], highlighting the possibility that exposure to micro- and nanoplastics may contribute to the onset or aggravation of neurodegenerative diseases.

Furthermore, inhibition of (A)ChE activity is among the most reported neurotoxic effects following exposure of bivalves, crustaceans and fish to micro- and nanoplastics (Table 1). Inhibition of (A)ChE activity is considered a reliable indicator for neuro (muscular) toxicity and is considered to disrupt (cholinergic) nervous system function once activity is inhibited by > 30% [115, 116]. Although there are some discrepancies and there is a general scarcity of in vivo mammalian data, most studies reported inhibition of (A)ChE of more than 30%, indicating a clear neurotoxic potential of micro- and nanoplastics. Importantly, inhibition of AChE has also been implicated in non-cholinergic functions related to neurite growth, synaptogenesis, cell migration, proliferation and apoptosis [116, 117]. However, whether or not exposure to micro- and nanoplastics affects these non-cholinergic functions remains to be determined.

Additional indications for the neurotoxic potential arise from the reported changes in dopamine levels in bivalves [71] and fish [86] after exposure to microplastics. Moreover, behavioral changes following exposure to microplastics have been reported for nematodes [69], crustaceans [77] and fish [22, 83, 85], although it should be noted that there are obviously considerable differences between the most-used species (such as fish and bivalves) and mammals including human with respect to physiology and routes of exposure (exposure via water (gills) vs. oral uptake (gut) or inhalation (lung)). Such differences in exposure routes may also affect uptake and/or distribution, thus hampering translation of effects observed in aquatic species to mammalian neurotoxic hazard characterization.

## Conclusions

**Fig. 1:**
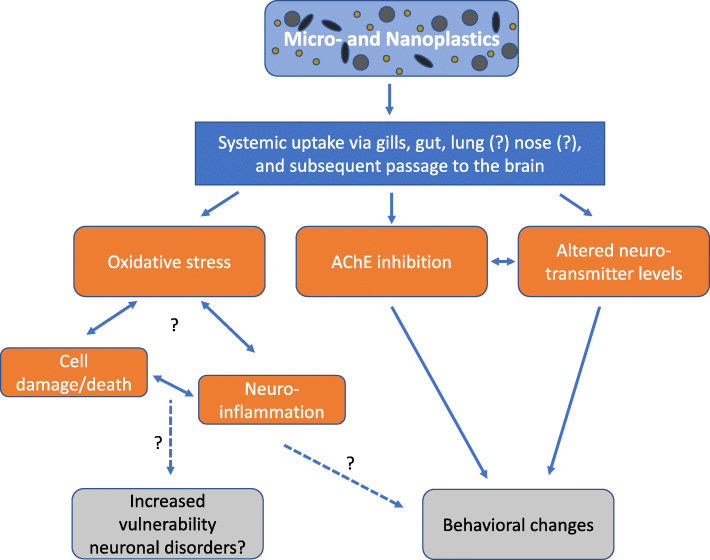
Overview of the neurotoxic effects of micro- and nanoplastics. Plastic particles can reach the systemic circulation and ultimately the brain via uptake through the gills, gut and possibly also the lungs or directly via the nasal cavity. Once in the brain, micro- and nanoplastics can induce oxidative stress, potentially resulting in cellular damage and neuroinflammation, which may ultimately increase onset and development of neurodevelopmental and/or neurodegenerative disorders. Micro- and nanoplastics in the brain can also results in inhibition of AChE and changes in neurotransmitter levels, which likely contribute to the observed behavioral changes. It should be noted though that most evidence is fragmentary and obtained from different, mainly aquatic species, highlighting the need for extensive systematic research to fully elucidate the neurotoxic potential of micro- and nanoplastics. See Table 1 for details

Despite the ubiquitous presence of micro- and nanoplastics in the environment, there is a general scarcity of data regarding their uptake and toxicity. Several studies have shown that micro- and nanoplastics are taken up via different exposure routes by various organisms, including fish and mammals. Despite the major knowledge gap with respect to the potential neurotoxicity of micro- and nanoplastics, these studies indicate that plastic particles can induce oxidative stress, inhibit AChE activity, alter neurotransmitter levels, and change behavior in several species (see Table 1 for details and Fig. 1 for a schematic overview). Whether these effects are related to (human) neurodevelopmental and/or neurodegenerative disorders, as shown for metal nanoparticles, remains to be determined.

Notably, most experimental exposures used so far are not very realistic for human exposure. Most studies used short exposure durations, with high exposure levels, while humans are chronically exposed to low levels. Additional shortcomings of the available studies include the use of (virgin) particle types and shapes that are not environmentally relevant. Moreover, a systematic comparison of different particle types, shapes, sizes and concentrations is lacking and to date most research focused on aquatic species.

Several actions are required to thoroughly elucidate the neurotoxic hazard and risk of exposure to micro- and nanoplastics. Firstly, exposure levels, in particularly for humans, require better monitoring. Emphasis should not only be on determining the level of exposure, but also the route of exposure (ingestion, inhalation and even retrograde transport following intranasal exposure) as well as particle characteristics (type, size, shape, weathering).

In parallel, exposure assessment should focus on the degree of uptake through the gut, lungs (or gills) or nasal epithelium, potential blood-brain barrier crossing, and potential translocation to or even accumulation in organs (including the brain). This will reveal whether or not particles translocate directly to the brain via olfactory and taste nerve endings, indirectly via the blood stream or both. Such information would help to determine whether atmospheric particles or food-based particles are most hazardous for human health. This would also provide indications for which exposure types mitigation measures would be most valuable.

Improvements of hazard characterization should involve standardizing the range of exposure times and particle doses to allow for dose- and time-response curves, also taking into account particle weight and number. Furthermore, different particle types, shapes, sizes and surface charges should be used, and these should preferably correspond with the plastic particles most abundant in the environment. Moreover, for a realistic risk assessment it would be beneficial to use aged and contaminated particles besides manufactured virgin particles, despite the resulting challenges regarding mixture toxicity.

Given the differences in exposure routes for different species, it is essential to use multiple species, including mammals. Notably, the required hazard characterization can to a large extend also be performed using in vitro assays, which may aid in increasing throughput, lowering costs, and increasing mechanistic insight. However, care should be taken to focus also on more subtle and functional endpoints and not only on overt (neuro)toxic endpoints, such as cell death, as these are likely only affected at non-realistic exposure levels. Regardless of the results of such hazard and risk assessment of micro- and nanoplastics, precautionary actions should be taken to minimize further contamination and spreading of macro-, micro- and nanoplastics into our environment.

## Supplementary information

**Additional file 1: Table S1.** Overview of the literature investigating neurotoxic effects of gold nanoparticles (Au-NPs). The reported particle size reflects the diameter of primary particles. Every study included either a control group that was not exposed to Au-NPs or any other substance, or pre-exposure measurements were taken as a control. **Table S2.** Overview of the literature investigating neurotoxic effects of titanium dioxide nanoparticles (TiO2-NPs). The reported particle size reflects the diameter of primary particles. Every study included a control group that was not exposed to TiO2 or any other substance, or pre-exposure measurements were taken as a control.

## Data Availability

Not applicable.

## Abbreviations

5-HT: Serotonin
AChE: Acetylcholinesterase
Au: Gold
BBB: Blood-brain barrier
BPA: Bisphenol-A
CAT: Catalase
CbE: Carboxylesterase
Cbz: Carbamazepine
Cd: Cadmium
ChE: Cholinesterase
DA: Dopamine
EE2: 17α-ethinylestradiol
Glu: Glutamate
GST: Glutathione-S-transferase
LPO: Lipid peroxidation
MAO: Monoamine oxidase
MDA: Malondialdehyde
MP: Microplastics
NP: Nanoplastics
PChE: Propionylcholinesterase
PE: Polyethylene
PET: Polyethylene terephthalate
PS: Polystyrene
ROS: Reactive oxygen species
ROX: Roxithromycin
SOD: Superoxide dismutase
TiO_2_: Titanium dioxide
ZonMW: The Netherlands Organisation for Health Research and Development

## References

[1] Alimba CG, Faggio C (2019). Microplastics in the marine environment: current trends in environmental pollution and mechanisms of toxicological profile. Environ Toxicol Pharmacol.

[2] Galgani F, Hanke G, Maes T, Bergmann M, Gutow L, Klages M (2015). Global Distribution, Composition and Abundance of Marine Litter. Marine Anthropogenic Litter.

[3] Chen CL, Bergmann M, Gutow L, Klages M (2015). Regulation and Management of Marine Litter. Marine Anthropogenic Litter.

[4] Eriksen M, Lebreton LCM, Carson HS, Thiel M, Moore CJ, Borerro JC (2014). Plastic pollution in the world’s oceans: more than 5 trillion plastic pieces weighing over 250,000 tons afloat at sea. PLoS One.

[5] Andrady AL (2017). The plastic in microplastics: a review. Mar Pollut Bull.

[6] Karbalaei S, Hanachi P, Walker TR, Cole M (2018). Occurrence, sources, human health impacts and mitigation of microplastic pollution. Environ Sci Pollut Res.

[7] Thompson RC, Olson Y, Mitchell RP, Davis A, Rowland SJ, John AW (2004). Lost at sea: where is all the plastic?. Science.

[8] Andrady AL, Bergmann M, Gutow L, Klages M (2015). Persistence of Plastic Litter in the Oceans. Marine Anthropogenic Litter.

[9] de Sá LC, Oliveira M, Ribeiro F, Rocha TL, Futter MN (2018). Studies of the effects of microplastics on aquatic organisms: what do we know and where should we focus our efforts in the future?. Sci Total Environ.

[10] Gerdes Z, Ogonowski M, Nybom I, Ek C, Adolfsson-Erici M, Barth A, Gorokhova E (2019). Microplastic-mediated transport of PCBs? A depuration study with Daphnia magna. PLoS One.

[11] Koelmans AA, Besseling E, Wegner A, Foekema EM (2013). Plastic as a carrier of POPs to aquatic organisms: a model analysis. Environ Sci Technol..

[12] Koelmans AA, Bakir A, Burton GA, Janssen CR (2016). Microplastic as a vector for chemicals in the aquatic environment: critical review and model-supported reinterpretation of empirical studies. Environ Sci Technol.

[13] Li J, Zhang K, Zhang H (2018). Adsorption of antibiotics on microplastics. Environ Pollut.

[14] Waring RH, Harris RM, Mitchell SC (2018). Plastic contamination of the food chain: a threat to human health?. Maturitas..

[15] Carbery M, O’Connor W, Palanisami T (2018). Trophic transfer of microplastics and mixed contaminants in the marine food web and implications for human health. Environ Int.

[16] Barboza LGA, Vieira LR, Branco V, Figueiredo N, Carvalho F, Carvalho C, Guilhermino L (2018). Microplastics cause neurotoxicity, oxidative damage and energy-related changes and interact with the bioaccumulation of mercury in the European seabass, Dicentrarchus labrax (Linnaeus, 1758). Aquat Toxicol.

[17] Su L, Deng H, Li B, Chen Q, Pettigrove V, Wu C, Shi H (2019). The occurrence of microplastic in specific organs in commercially caught fishes from coast and estuary area of East China. J Hazard Mater.

[18] Wright SL, Kelly FJ (2017). Plastic and human health: a micro issue?. Environ Sci Technol.

[19] Sharma S, Chatterjee S (2017). Microplastic pollution, a threat to marine ecosystem and human health: a short review. Environ Sci Pollut Res.

[20] Browne MA, Dissanayake A, Galloway TS, Lowe DM, Thompson RC (2008). Ingested microscopic plastic translocates to the circulatory system of the mussel, Mytilus edulis (L.). Environ Sci Technol.

[21] Cui R, Kim SW, An YJ (2017). Polystyrene nanoplastics inhibit reproduction and induce abnormal embryonic development in the freshwater crustacean Daphnia galeata. Sci Rep.

[22] Mattsson K, Johnson EV, Malmendal A, Linse S, Hansson LA, Cedervall T (2017). Brain damage and behavioural disorders in fish induced by plastic nanoparticles delivered through the food chain. Sci Rep.

[23] Bouwmeester H, Hollman PCH, Peters RJB (2015). Potential health impact of environmentally released micro- and nanoplastics in the human food production chain: experiences from nanotoxicology. Environ Sci Technol.

[24] Kole PJ, Löhr AJ, Van Belleghem F, Ragas A (2017). Wear and tear of tyres: a stealthy source of microplastics in the environment. Int J Environ Res Public Health.

[25] Toussaint B, Raffael B, Angers-Loustau A, Gilliland D, Kestens V, Petrillo M (2019). Review of micro-and nanoplastic contamination in the food chain. Food Additives & Contaminants: Part A.

[26] Pivokonsky M, Cermakova L, Novotna K, Peer P, Cajthaml T, Janda V (2018). Occurrence of microplastics in raw and treated drinking water. Sci Total Environ.

[27] Oßmann BE, Sarau G, Holtmannspötter H, Pischetsrieder M, Christiansen SH, Dicke W (2018). Small-sized microplastics and pigmented particles in bottled mineral water. Water Res.

[28] Prata JC (2018). Airborne microplastics: consequences to human health?. Environ Pollut.

[29] Jani P, Halbert GW, Langridge J, Florence AT (1990). Nanoparticle uptake by the rat gastrointestinal mucosa: quantitation and particle size dependency. J Pharm Pharmacol.

[30] Stock V, Bohmert L, Lisicki E, Block R, Cara-Carmona J, Pack LK (2019). Uptake and effects of orally ingested polystyrene microplastic particles in vitro and in vivo. Arch Toxicol.

[31] Pauly JL, Stegmeier SJ, Allaart HA, Cheney RT, Zhang PJ, Mayer AG, Streck RJ (1998). Inhaled cellulosic and plastic fibers found in human lung tissue. Cancer Epidemiol Biomarkers Prev.

[32] Schmidt C, Lautenschlaeger C, Collnot EM, Schumann M, Bojarski C, Schulzke JD (2013). Nano- and microscaled particles for drug targeting to inflamed intestinal mucosa - a first in vivo study in human patients. J Control Release.

[33] Vethaak DA, Leslie HA (2016). Plastic debris is a human health issue. Environ Sci Technol..

[34] Rubio L, Marcos R, Hernández A (2020). Potential adverse health effects of ingested micro-and nanoplastics on humans. Lessons learned from in vivo and in vitro mammalian models. J Toxicol Environ Health Part B.

[35] Lehner R, Weder C, Petri-Fink A, Rothen-Rutishauser B (2019). Emergence of nanoplastic in the environment and possible impact on human health. Environ Sci Technol.

[36] Prata JC, da Costa JP, Lopes I, Duarte AC, Rocha-Santos T (2019). Environmental exposure to microplastics: An overview on possible human health effects. Sci Total Environ.

[37] Singh R, Lillard JW (2009). Nanoparticle-based targeted drug delivery. Exp Mol Pathol.

[38] Win-Shwe TT, Fujimaki H (2011). Nanoparticles and neurotoxicity. Int J Mol Sci.

[39] Khan FA, Almohazey D, Alomari M, Almofty SA (2018). Impact of nanoparticles on neuron biology: current research trends. Int J Nanomedicine.

[40] Karmakar A, Zhang Q, Zhang Y (2014). Neurotoxicity of nanoscale materials. J Food Drug Anal.

[41] Teleanu D, Chircov C, Grumezescu A, Teleanu R, Teleanu DM, Chircov C (2019). Neurotoxicity of nanomaterials: An up-to-date overview. Nanomaterials..

[42] Oszlánczi G, Vezér T, Sárközi L, Horváth E, Szabó A, Horváth E (2010). Metal deposition and functional neurotoxicity in rats after 3-6 weeks nasal exposure by two physicochemical forms of manganese. Environ Toxicol Pharmacol.

[43] Borisova T (2018). Nervous system injury in response to contact with environmental, engineered and planetary micro- and nano-sized particles. Front Physiol.

[44] Boyes WK, van Thriel C. Neurotoxicology of Nanomaterials. Chemical research in toxicology. 2020. Ahead of print. 10.1021/acs.chemrestox.0c00050. 10.1021/acs.chemrestox.0c00050 PMC8293923 32233399

[45] Wu J, Ding T, Sun J (2013). Neurotoxic potential of iron oxide nanoparticles in the rat brain striatum and hippocampus. Neurotoxicology..

[46] Fernández-Bertólez N, Costa C, Bessa MJ, Park M, Carriere M, Dussert F (2019). Assessment of oxidative damage induced by iron oxide nanoparticles on different nervous system cells. Mutat Res.

[47] Haase A, Rott S, Mantion A, Graf P, Plendl J, Thünemann AF (2012). Effects of silver nanoparticles on primary mixed neural cell cultures: uptake, oxidative stress and acute calcium responses. Toxicol Sci.

[48] Liu Y, Guan W, Ren G, Yang Z (2012). The possible mechanism of silver nanoparticle impact on hippocampal synaptic plasticity and spatial cognition in rats. Toxicol Lett.

[49] Niska K, Santos-Martinez MJ, Radomski MW, Inkielewicz-Stepniak I (2015). CuO nanoparticles induce apoptosis by impairing the antioxidant defense and detoxification systems in the mouse hippocampal HT22 cell line: protective effect of crocetin. Toxicol in Vitro.

[50] An L, Liu S, Yang Z, Zhang T (2012). Cognitive impairment in rats induced by nano-CuO and its possible mechanisms. Toxicol Lett.

[51] Luyts K, Napierska D, Nemery B, Hoet PHM (2013). How physico-chemical characteristics of nanoparticles cause their toxicity: complex and unresolved interrelations. Environ Sci Processes Impacts.

[52] Truong L, Saili KS, Miller JM, Hutchison JE, Tanguay RL (2012). Persistent adult zebrafish behavioral deficits results from acute embryonic exposure to gold nanoparticles. Comp Biochem Physiol C Toxicol Pharmacol.

[53] Dedeh A, Ciutat A, Treguer-Delapierre M, Bourdineaud JP (2015). Impact of gold nanoparticles on zebrafish exposed to a spiked sediment. Nanotoxicology..

[54] Ferreira GK, Cardoso E, Vuolo FS, Galant LS, Michels M, Gonçalves CL (2017). Effect of acute and long-term administration of gold nanoparticles on biochemical parameters in rat brain. Mater Sci Eng C.

[55] Miranda RR, Damaso da Silveira ALR, de Jesus IP, Grötzner SR, Voigt CL, Campos SX (2016). Effects of realistic concentrations of TiO 2 and ZnO nanoparticles in Prochilodus lineatus juvenile fish. Environ Sci Pollut Res.

[56] Sheng L, Wang L, Su M, Zhao X, Hu R, Yu X (2016). Mechanism of TiO2 nanoparticle-induced neurotoxicity in zebrafish (Danio rerio). Environ Toxicol.

[57] Hu Q, Guo F, Zhao F, Fu Z (2017). Effects of titanium dioxide nanoparticles exposure on parkinsonism in zebrafish larvae and PC12. Chemosphere..

[58] Carmo TLL, Siqueira PR, Azevedo VC, Tavares D, Pesenti EC, Cestari MM (2019). Overview of the toxic effects of titanium dioxide nanoparticles in blood, liver, muscles, and brain of a Neotropical detritivorous fish. Environ Toxicol.

[59] Shrivastava R, Raza S, Yadav A, Kushwaha P, Flora SJS (2014). Effects of sub-acute exposure to TiO_2_, ZnO and Al_2_O_3_ nanoparticles on oxidative stress and histological changes in mouse liver and brain. Drug Chem Toxicol.

[60] Grissa I, Guezguez S, Ezzi L, Chakroun S, Sallem A, Kerkeni E (2016). The effect of titanium dioxide nanoparticles on neuroinflammation response in rat brain. Environ Sci Pollut Res.

[61] Hu R, Gong X, Duan Y, Li N, Che Y, Cui Y (2010). Neurotoxicological effects and the impairment of spatial recognition memory in mice caused by exposure to TiO_2_ nanoparticles. Biomaterials..

[62] Ze X, Su M, Zhao X, Jiang H, Hong J, Yu X (2014). TiO2nanoparticle-induced neurotoxicity may be involved in dysfunction of glutamate metabolism and its receptor expression in mice. Environ Toxicol.

[63] Horváth T, Vezér T, Kozma G, Papp A (2018). Functional neurotoxicity and tissue metal levels in rats exposed subacutely to titanium dioxide nanoparticles via the airways. Ideggyogy Sz.

[64] Ze Y, Sheng L, Zhao X, Ze X, Wang X, Zhou Q (2013). Neurotoxic characteristics of spatial recognition damage of the hippocampus in mice following subchronic peroral exposure to TiO2 nanoparticles. J Hazard Mater.

[65] Long TC, Saleh N, Tilton RD, Lowry GV, Veronesi B (2006). Titanium dioxide (P25) produces reactive oxygen species in immortalized brain microglia (BV2): implications for nanoparticle neurotoxicity. Environ Sci Technol.

[66] Coccini T, Grandi S, Lonati D, Locatelli C, De Simone U (2015). Comparative cellular toxicity of titanium dioxide nanoparticles on human astrocyte and neuronal cells after acute and prolonged exposure. NeuroToxicology..

[67] Erriquez J, Bolis V, Morel S, Fenoglio I, Fubini B, Quagliotto P, Distasi C (2015). Nanosized TiO2 is internalized by dorsal root ganglion cells and causes damage via apoptosis. Nanomedicine.

[68] He Q, Zhou X, Liu Y, Gou W, Cui J, Li Z (2018). Titanium dioxide nanoparticles induce mouse hippocampal neuron apoptosis via oxidative stress- and calcium imbalance-mediated endoplasmic reticulum stress. Environ Toxicol Pharmacol.

[69] Lei L, Liu M, Song Y, Lu S, Hu J, Cao C (2018). Polystyrene (nano) microplastics cause size-dependent neurotoxicity, oxidative damage and other adverse effects in Caenorhabditis elegans. Environmental Science: Nano.

[70] Chen Y, Liu X, Leng Y, Wang J. Defense responses in earthworms (Eisenia fetida) exposed to low-density polyethylene microplastics in soils. Ecotoxicol Environ Saf. 2020;187109788. 10.1016/j.ecoenv.2019.109788. 10.1016/j.ecoenv.2019.109788 31648073

[71] Magni S, Gagné F, André C, Della Torre C, Auclair J, Hanana H (2018). Evaluation of uptake and chronic toxicity of virgin polystyrene microbeads in freshwater zebra mussel Dreissena polymorpha (Mollusca: Bivalvia). Sci Total Environ.

[72] Ribeiro F, Garcia AR, Pereira BP, Fonseca M, Mestre NC, Fonseca TG (2017). Microplastics effects in Scrobicularia plana. Mar Pollut Bull.

[73] Brandts I, Teles M, Gonçalves AP, Barreto A, Franco-Martinez L, Tvarijonaviciute (2018). Effects of nanoplastics on Mytilus galloprovincialis after individual and combined exposure with carbamazepine. Sci Total Environ.

[74] Avio CG, Gorbi S, Milan M, Benedetti M, Fattorini D, D’Errico G (2015). Pollutants bioavailability and toxicological risk from microplastics to marine mussels. Environ Pollut.

[75] Guilhermino L, Vieira LR, Ribeiro D, Tavares AS, Cardoso V, Alves A, Almeida J (2018). Uptake and effects of the antimicrobial florfenicol, microplastics and their mixtures on freshwater exotic invasive bivalve Corbicula fluminea. Sci Total Environ.

[76] Oliveira P, Barboza LGA, Branco V, Figueiredo N, Carvalho C, Guilhermino L (2018). Effects of microplastics and mercury in the freshwater bivalve Corbicula fluminea (Müller, 1774): filtration rate, biochemical biomarkers and mercury bioconcentration. Ecotoxicol Environ Saf.

[77] Gambardella C, Morgana S, Ferrando S, Bramini M, Piazza V, Costa E (2017). Effects of polystyrene microbeads in marine planktonic crustaceans. Ecotoxicol Environ Saf.

[78] Varó I, Perini A, Torreblanca A, Garcia Y, Bergami E, Vannuccini ML, Corsi I (2019). Time-dependent effects of polystyrene nanoparticles in brine shrimp Artemia franciscana at physiological, biochemical and molecular levels. Sci Total Environ.

[79] Kashiwada S (2006). Distribution of nanoparticles in the see-through medaka (Oryzias latipes). Environ Health Perspect.

[80] Ding J, Zhang S, Razanajatovo RM, Zou H, Zhu W (2018). Accumulation, tissue distribution, and biochemical effects of polystyrene microplastics in the freshwater fish red tilapia (Oreochromis niloticus). Environ Pollut.

[81] Jacob H, Gilson A, Lanctôt C, Besson M, Metian M, Lecchini D (2019). No effect of polystyrene microplastics on foraging activity and survival in a post-larvae coral-reef fish, Acanthurus triostegus. Bull Environ Contam Toxicol.

[82] Karami A, Groman DB, Wilson SP, Ismail P, Neela VK (2017). Biomarker responses in zebrafish (Danio rerio) larvae exposed to pristine low-density polyethylene fragments. Environ Pollut.

[83] Mak CW, Ching-Fong Yeung K, Chan KM (2019). Acute toxic effects of polyethylene microplastic on adult zebrafish. Ecotoxicol Environ Saf.

[84] Wen B, Zhang N, Jin SR, Chen ZZ, Gao JZ, Liu Y (2018). Microplastics have a more profound impact than elevated temperatures on the predatory performance, digestion and energy metabolism of an Amazonian cichlid. Aquat Toxicol.

[85] Chen Q, Gundlach M, Yang S, Jiang J, Velki M, Yin D, Hollert H (2017). Quantitative investigation of the mechanisms of microplastics and nanoplastics toward zebrafish larvae locomotor activity. Sci Total Environ.

[86] Chen Q, Yin D, Jia Y, Schiwy S, Legradi J, Yang S, Hollert H (2017). Enhanced uptake of BPA in the presence of nanoplastics can lead to neurotoxic effects in adult zebrafish. Sci Total Environ.

[87] Guven O, Bach L, Munk P, Dinh KV, Mariani P, Nielsen TG (2018). Microplastic does not magnify the acute effect of PAH pyrene on predatory performance of a tropical fish (Lates calcarifer). Aquat Toxicol.

[88] Zhang S, Ding J, Razanajatovo RM, Jiang H, Zou H, Zhu W (2019). Interactive effects of polystyrene microplastics and roxithromycin on bioaccumulation and biochemical status in the freshwater fish red tilapia (Oreochromis niloticus). Sci Total Environ.

[89] Luís LG, Ferreira P, Fonte E, Oliveira M, Guilhermino L (2015). Does the presence of microplastics influence the acute toxicity of chromium (VI) to early juveniles of the common goby (Pomatoschistus microps)? A study with juveniles from two wild estuarine populations. Aquat Toxicol.

[90] Miranda T, Vieira LR, Guilhermino L (2019). Neurotoxicity, behavior, and lethal effects of cadmium, microplastics and their mixtures on Pomatoschistus microps juveniles from two wild populations exposed under laboratory conditions — Implications to environmental and human risk assessment. Int J Environ Res Public Health..

[91] Oliveira M, Ribeiro A, Hylland K, Guilhermino L (2013). Single and combined effects of microplastics and pyrene on juveniles (0+ group) of the common goby Pomatoschistus microps (Teleostei, Gobiidae). Ecol Indic.

[92] Ferreira P, Fonte E, Soares ME, Carvalho F, Guilhermino L (2016). Effects of multi-stressors on juveniles of the marine fish Pomatoschistus microps: gold nanoparticles, microplastics and temperature. Aquat Toxicol.

[93] Fonte E, Ferreira P, Guilhermino L (2016). Temperature rise and microplastics interact with the toxicity of the antibiotic cefalexin to juveniles of the common goby (Pomatoschistus microps): post-exposure predatory behaviour, acetylcholinesterase activity and lipid peroxidation. Aquat Toxicol.

[94] Deng Y, Zhang Y, Lemos B, Ren H (2017). Tissue accumulation of microplastics in mice and biomarker responses suggest widespread health risks of exposure. Sci Rep.

[95] Rafiee M, Dargahi L, Eslami A, Beirami E, Jahangiri-rad M, Sabour S, Amereh F (2018). Neurobehavioral assessment of rats exposed to pristine polystyrene nanoplastics upon oral exposure. Chemosphere..

[96] Schirinzi GF, Pérez-Pomeda I, Sanchís J, Rossini C, Farré M, Barceló D (2017). Cytotoxic effects of commonly used nanomaterials and microplastics on cerebral and epithelial human cells. Environ Res.

[97] Murali K, Kenesei K, Li Y, Demeter K, Környei Z, Madarász E (2015). Uptake and bio-reactivity of polystyrene nanoparticles is affected by surface modifications, ageing and LPS adsorption: in vitro studies on neural tissue cells. Nanoscale..

[98] Hoelting L, Scheinhardt B, Bondarenko O, Schildknecht S, Kapitza M, Tanavde V (2013). A 3-dimensional human embryonic stem cell (hESC)-derived model to detect developmental neurotoxicity of nanoparticles. Arch Toxicol.

[99] Jin Y, Lu L, Tu W, Luo T, Fu Z (2019). Impacts of polystyrene microplastic on the gut barrier, microbiota and metabolism of mice. Sci Total Environ.

[100] Yong CQY, Valiyaveetill S, Tang BL (2020). Toxicity of microplastics and Nanoplastics in mammalian systems. Int J Environ Res Public Health.

[101] Wang YL, Lee YH, Chiu IJ, Lin YF, Chiu HW (2020). Potent impact of plastic nanomaterials and micromaterials on the food chain and human health. Int J Mol Sci.

[102] World Health Organization. (2019). Microplastics in drinking-water. Retrieved from https://www.who.int/water_sanitation_health/publications/microplastics-in-drinking-water/en/. Accessed 3 Feb 2020.

[103] Patil S, Sandberg A, Heckert E, Self W, Seal S (2007). Protein adsorption and cellular uptake of cerium oxide nanoparticles as a function of zeta potential. Biomaterials..

[104] Fröhlich E (2012). The role of surface charge in cellular uptake and cytotoxicity of medical nanoparticles. Int J Nanomedicine.

[105] Virsek MK, Lovsin MN, Koren S, Krzan A, Peterlin M (2017). Microplastics as a vector for the transport of the bacterial fish pathogen species Aeromonas salmonicida. Mar Pollut Bull.

[106] Koelmans AA, Mohamed Nor NH, Hermsen E, Kooi M, Mintenig SM, De France J (2019). Microplastics in freshwaters and drinking water: critical review and assessment of data quality. Water Res.

[107] Hermsen E, Mintenig SM, Besseling E, Koelmans AA (2018). Quality criteria for the analysis of microplastic in biota samples: a critical review. Environ Sci Technol.

[108] van Raamsdonk LWD, van der Zande M, Koelmans AA, Hoogenboom RLAP, Peters RJB, Groot MJ (2020). Current insights into monitoring, bioaccumulation, and potential health effects of microplastics present in the food chain. Foods..

[109] Smith M, Love DC, Rochman CM, Neff RA (2018). Microplastics in seafood and the implications for human health. Curr Environ Health Rep.

[110] Yang CS, Chang CH, Tsai PJ, Chen WY, Tseng FG, Lo LW (2004). Nanoparticle-based in vivo investigation on blood-brain barrier permeability following ischemia and reperfusion. Anal Chem.

[111] Zauner W, Farrow NA, Haines AM (2001). In vitro uptake of polystyrene microspheres: effect of particle size, cell line and cell density. J Control Release.

[112] Kulkarni SA, Feng SS (2013). Effects of particle size and surface modification on cellular uptake and biodistribution of polymeric nanoparticles for drug delivery. Pharm Res.

[113] Tönnies E, Trushina E (2017). Oxidative stress, synaptic dysfunction, and Alzheimer’s disease. J Alzheimers Dis.

[114] Radi E, Formichi P, Battisti C, Federico A (2014). Apoptosis and oxidative stress in neurodegenerative diseases. J Alzheimers Dis.

[115] Almeida JR, Oliveira C, Gravato C, Guilhermino L (2010). Linking behavioural alterations with biomarkers responses in the european seabass dicentrarchus labrax l. exposed to the organophosphate pesticide fenitrothion. Ecotoxicology..

[116] Lionetto MG, Caricato R, Calisi A, Giordano ME, Schettino T (2013). Acetylcholinesterase as a biomarker in environmental and occupational medicine: new insights and future perspectives. Biomed Res Int.

[117] Lazarevic-Pasti T, Leskovac A, Momic T, Petrovic S, Vasic V (2017). Modulators of acetylcholinesterase activity: from Alzheimer’s disease to anti-cancer drugs. Curr Med Chem.

