# Supplementary material for: The plastic brain: neurotoxicity of micro- and nanoplastics
Source: Part Fibre Toxicol. 2020 Jun 8;17:24. doi: 10.1186/s12989-020-00358-y (PMC7282048; doi:10.1186/s12989-020-00358-y)
Supplement: Supplementary file 1 — Additional file 1: Table S1. Overview of the literature investigating neurotoxic effects of gold nanoparticles (Au-NPs). The reported particle size reflects the diameter of primary particles. Every study included either a control group that was not exposed to Au-NPs or any other substance, or pre-exposure measurements were taken as a control. Table S2. Overview of the literature investigating neurotoxic effects of titanium dioxide nanoparticles (TiO2-NPs). The reported particle size reflects the diameter of primary particles. Every study included a control group that was not exposed to TiO2 or any other substance, or pre-exposure measurements were taken as a control. [file 12989_2020_358_MOESM1_ESM.pdf]

The plastic brain: neurotoxicity of micro- and nanoplastics

Supplemental materials: Neurotoxicity of chemically inert metal nanoparticles

Minne Prüst, Jonelle Meijer, Remco HS Westerink*

Neurotoxicology Research Group, Division Toxicology, Institute for Risk Assessment Sciences (IRAS), Faculty of Veterinary Medicine, Utrecht University, NL-3508 TD, Utrecht, The Netherlands

* corresponding author; r.westerink@uu.nl

Literature search

A Pubmed search was performed in August 2019 using the following combinations of search words: "Neurotox* AND nanoparticles AND gold" (47 hits) and "Neurotox* AND nanoparticles AND titanium dioxide" (60 hits). Publications directed at investigating the neurotoxicity of Au- or TiO_2-nanoparticles (alone or co-exposed with other compounds) on animals, neuronal cells and organelles were included in the literature analysis. Publications were excluded from the analysis if the research involved metal(oxide) nanoparticles coated with other compounds or antibodies severely altering physiochemical properties of the nanoparticles, or if metal(oxide) nanoparticles were used solely for non-related purposes such as imaging, pesticide sensing, water purification or as carriers in personalized medicine or therapy. In total 53 publications were selected for Au-nanoparticles (7 publications) and TiO_2-nanoparticles (46 publications). No additional publications were found via the bibliography of these publications.

Seven publications investigating the neurotoxicity of Au-nanoparticles were selected (**Table S1**). Three of the studies used zebrafish as model organism, one study used adult male rats and three studies used human and animal-derived neurons or neuronal endothelial cells *in vitro*. The studied Au particles had a maximum primary particle size of 60 nm and thus were all classified as nanoparticles. Five studies used regular Au-nanoparticles and one study also investigated bulk Au-nanoparticles. Two studies used coated Au-nanoparticles; these coatings did not alter other physiochemical properties of the particle except surface charge. None of the selected publications on the neurotoxic effects of Au-nanoparticles involved co-exposure with other compounds.

In total, 46 publications investigating the neurotoxicity of TiO_2-nanoparticles were selected (**Table S2**). Seventeen articles described the use of rodents (mice and rats, both adult, adolescent and prenatal), sixteen studies conducted research on neuronal cell *in vitro*, eight studies used fish (adults and embryos). Five studies used marine invertebrates (organism or gametes), amongst which: Annelida (1), Echinodermata (1), bivalves (2) and planktonic crustaceans (1). One study investigated the toxicity of TiO_2-NPs on isolated brain mitochondria derived from male rats. All studies used nanosized TiO_2 particles, although some additionally used microsized particles. The diameter of the particles used in the experiment ranged from 5 nm to 1 µm. Different mineral structures of TiO_2 particles were investigated: anatase, rutile or a mixture of anatase and rutile. The crystal structure of TiO_2-NPs influences the physicochemical properties and the utility of the particles. The anatase structure is most studied, and most studies only investigated TiO_2 particles of one structure, although a few studies compared toxicity of the different structures. Several of the selected publications on the neurotoxic effects of TiO_2-nanoparticles involved co-exposure with other compounds, such as cypermethrin, lead, polybrominated diphenyl ether, zinc oxide, zinc dioxide, aminoguanidine or other metal(oxide) nanoparticles.

Table S1 – *Overview of the literature investigating neurotoxic effects of gold nanoparticles (Au-NPs). The reported particle size reflects the diameter of primary particles. Every study included either a control group that was not exposed to Au-NPs or any other substance, or pre-exposure measurements were taken as a control.*

Abbreviations: Au-NPs: gold nanoparticles, AChE: acetylcholine esterase; BBB: blood-brain barrier; GSH: glutathione; MEE(E): 2-(2-(2-mercaptoethoxy) ethoxy) ethanol (neutral surface charge); MES: mercaptoethanesulfonic acid (negative surface charge); NPs: nanoparticles; SOD: superoxide dismutase; TMAT: trimethylammonium methanethiol (positive surface charge).

Model system	Particle type / size	Exposure method	Exposure dose	(Neuro)toxic effects	Ref.
**Fish**					
Zebrafish (*Danio rerio)*	Au-NPs of 14 nm	For 20 days; exposure via water	0.25 µg/L<br>0.8 µg/L	Oxidative stress in brain, mitochondrial metabolism detoxification and altered neurotransmission (0.8 µg/L); increased AChE activity (0.25 µg/L and 0.8 µg/L); no accumulation in brain tissue.	[1]
Zebrafish embryos (*Danio rerio*)	Au-NPs of 0.8, 1.5 and 15 nm with negative (MES), positive (TMAT) or neutral (MEE(E)) surface charge	For 24, 48 and 120 hours post-fertilization via embryo medium	16, 80, 400 ppb<br>2, 10, 50, 250 ppm	Increased mortality and malformation (≥ 80 ppb TMAT, ≥ 400 pb MES); no explicit behavioral alternations; no changes after exposure with non-charged Au-NPs.	[2]
Zebrafish embryos and adults (*Danio rerio*)	Au-NPs of 1.5 nm, with negative (MES), positive (TMAT) or neutral (MEEE) surface charge	For 6 and 120 hours post-fertilization via embryo medium	50 µg/L (MES)<br>10 µg/L (MEEE and TMAT)	Locomotor hypoactivity and behavioral irregularities during embryonic stage (MES and TMAT); abnormal startle behavior in adult zebrafish (MES and TMAT); reduced survival after maturation (MES).	[3]
**Mammals**					
Adult male Wistar rats	Au-NPs of 10 and 30 nm	Once (acute) and daily for 28 days (chronic) intraperitoneal injection	70 µg/kg	Uptake of Au-NPs (both 10 and 30 nm) in brain (chronic ≥ acute); inhibition of energy metabolism in cerebral cortex (acute 10 nm, chronic 10 and 30 nm); induction of oxidative stress (SOD).	[4]

***In vitro* cell culture**					
N9 Microglial cells and human SH-SY5Y neuroblastoma cells	Au-NPs of 60 nm	For 6, 12 and 24 hours; via cell culture medium	10^3 and 2.6 x 10^9 NPs/mL	Uptake by cells in cytoplasm and vacuoles (2.6 x 10^9); increased oxidative stress (GSH) (6 and 24 hours, 2.6 x 10^3 and 2.6 x 10^9); no cytotoxicity; no change in cell viability; unaltered cell apoptosis biomarkers.	[5]
Rodent brain micro vessel endothelial cells	Au-NPs of 3, 5, 7, 10, 30 and 60 nm	For 2,4 and 8 hours; via cell culture medium	50 µg/mL	Significant decrease in cell viability (3 nm); insignificant change in BBB permeability; no alterations in inflammatory biomarkers.	[6]
Porcine brain micro vessel endothelial cells	Au-NPs of 3 and 5 nm	For 8-12 days; via cell culture medium	15 µg/mL	No significant change in extracellular pro-inflammatory mediators (Au-NPs); no significant alteration in permeability of BBB model.	[7]

Table S2 – Overview of the literature investigating neurotoxic effects of titanium dioxide nanoparticles (TiO_2-NPs). The reported particle size reflects the diameter of primary particles. Every study included a control group that was not exposed to TiO_2 or any other substance, or pre-exposure measurements were taken as a control.

Abbreviations: α-syn; α-synuclein; 5-HT: serotonin; AChE: acetylcholine esterase; AG: aminoguanidine; Ag: silver; A-NPs: anatase NPs; A/R-NPs: anatase-rutile NPs; Au: gold; BBB: blood-brain barrier; BDE: brominated diphenyl ether; CAT: catalase; Cyp: cypermethrin; DA: dopamine; GABA: gamma-aminobutyric acid; Glu: glutamate; GSH: glutathione; GST: glutathione-S-transferase; IL-6: interleukin 6; IP: intraperitoneal; IV: intravenous; LPO: lipid peroxidation; MDA: malondialdehyde; MPs: microparticles; NA: noradrenaline; NO: nitric oxide; NPs: nanoparticles; Pb: lead; R-NPs: rutile NPs; ROS: reactive oxygen species; Si: silica; SOD: superoxide dismutase; TiO_2-NPs: titanium dioxide nanoparticles; ZnO: zinc oxide.

Model system	Particle type / size	Exposure method	Exposure dose	(Neuro)toxic effects	Ref.
**Marine invertebrates**					
Earth worm (*Pheretima hawayana*)	TiO_2-NPs of 20-40 nm	For 3, 14, 28 days exposure via sediment	10, 50, 100 µg/kg	Decreased AChE activity (dose-dependent) (3 and 14 days > 28 days, ≥ 10 µg/kg); increase of lipid peroxidation (MDA) (14 days, 50 µg/kg and 28 days, 10 µg/kg).	[8]
Sea urchin gametes and developing embryos (*Paracentrotus lividus)*	TiO_2-NPs of 10-30 nm	For 1 hour to male gametes via water	0.1, 1, 10, 100 and 1000 µg/L	Decreased AChE activity (1 µg/L TiO_2).	[9]
Mediterranean clam *(Ruditapes decussatus)*	TiO_2-NPs of 10-15 nm	For 14 days; exposure via water	50 and 100 µg/L	Decreased AChE activity in gills (TiO_2-NPs 50 and 100 µg/L) and digestive gland (TiO_2-NPs 50 and 100 µg/L).	[10]
Chinese scallop (*Chlamys farreri*)	TiO_2-NPs of 21 nm	For 1-14 days; exposure via water	1 mg/L	Increased AChE activity in gills (≥ 5 days) and digestive gland (≥ 12 days); increased lipid peroxidation (MDA) (≥ 3 days).	[11]
Planktonic crustacean (*Daphnia magna*)	TiO_2-NPs of 60 nm, co-exposure with UV-Light and natural organic matter	For 96 hours; exposure via water	8 mg/L	Increased AChE activity (TiO_2 with high UV intensity); attenuated effect in presence with organic matter.	[12]

Fish					
Zebrafish embryos (*Danio rerio*)	TiO_2-NPs of 33 nm	For 96 hours post-fertilization via water	0.1, 1 and 10 µg/mL	Accumulation of TiO_2 particles in brain (≥ 1 µg/mL); increased ROS generation (≥ 1 µg/mL); cell death in hypothalamus (≥ 10 µg/mL); increased expression of dementia related genes (≥ 1 µg/mL); loss of dopaminergic neurons (≥ 0.1 µg/mL); no increase in mortality.	[13]
Zebrafish embryos (*Danio rerio*)	TiO_2-NPs of 21 nm	For 72 hours post-fertilization via water	1 mg/L	No differences in mortality or hatching rate; normal (neuro)development; no alterations in microglia distribution.	[14]
Zebrafish embryos (*Danio rerio*)	TiO_2-NPs of 7 nm, co-exposure with Cyp	For 120 hours post-fertilization via water	1 mg/L TiO_2 (0.4, 2 and 10 µg/L Cyp)	Enhanced neurotoxic effects of Cyp and accumulation of TiO_2; decreased locomotor activity; decreased neurotransmitter levels (DA, 5-HT, GABA); decreased gene expression important for (neuro)development.	[15]
Zebrafish embryos (*Danio rerio*)	TiO_2-NPs of 7 nm co-exposure with Pb	For 6 days post-fertilization via water	0.1 mg/L TiO_2 (5, 10, 20 and 30 µg/L Pb)	Decreased gene expression for (neuro)development; enhanced locomotion activity.	[16]
Zebrafish embryos (*Danio rerio*)	TiO_2-NPs of 7 nm co-exposure with BDE	For 7 days post-fertilization via water	0.1 mg/L TiO_2 (0.38 mg/L BDE)	Absorption of BDE by TiO_2-NPs; enhanced accumulation and neurotoxicity (mixture compared to BDE alone); no oxidative stress (ROS or LPO); no developmental toxicity.	[17]
Adult zebrafish (*Danio rerio*)	TiO_2-NPs of 33 nm	For 45 days (sub-chronic) via water	5, 10, 20 and 40 µg/L	Impaired spatial recognition memory; altered locomotor behavior (≥ 5 µg/L); decreased brain weight and accumulation of NP in brain, heart and liver (≥ 10 µg/L); decreased levels of NA, DA and 5-HT (≥ 10 µg/L); neuron apoptosis and over-proliferation of glial cells (low doses); mitochondrial swelling in neurons (≥ 5 µg/L); accumulation of NPs in cytoplasm or nuclei of neural cells (≥ 5 µg/L).	[18]

Juvenile streaked prochilod (*Prochilodus lineatus)*	TiO_2-NPs of 21 nm	For 48 hours (acute) and 14 days (sub-chronic) via water	1, 5, 10 and 50 mg/L	TiO_2-NP accumulation in brain, liver and muscle (≥ 10 mg/L, 14 days); decreased muscular AChE activity (1, 5 and 50 mg/L, 48 hours); ROS generation (≥ 5 mg/L, 48 hours); increased GST activity (≥ 10 mg/L, 14 days); no decrease of AChE in brain; no alterations in locomotor activity and behavior.	[19]
Juvenile streaked prochilod (*Prochilodus lineatus)*	TiO_2-NPs of 21 nm, alone and co-exposure with ZnO-NPs of 70 nm	For 5 and 30 days (sub-chronic) via water	0.1, 1 and 10 µg/L (7, 70 and 700 µg/mL ZnO)	Decreased muscular AChE activity (10 µg/L, 5 days); increased AChE activity (1 µg/L TiO_2 and 70 µg/mL ZnO mixture, 30 days); possible accumulation of TiO_2-NPs in axons of the inner retina (0.1 µg/L TiO_2, 5 and 30 days); no mortality (all concentrations).	[20]
**Mammals**					
Mouse neonates CD-1 strain (*Mus musculus*)	TiO_2-NPs of 6.5 nm	Maternal oral insulation Daily from prenatal day 0 to postnatal day 21	1, 2 and 3 mg/kg	Dose-dependent impairment of learning and memory (≥ 1 mg/kg); thinning of cerebral and cerebellar cortex (≥ 1 mg/kg); changes in hippocampal neurons (≥ 1 mg/kg); decreased neurite outgrowth and neuron morphology alterations (≥ 1 mg/kg); genotoxicity of neurodevelopment genes (≥ 1 mg/kg).	[21]
Mouse neonates NMRI strain (*Mus musculus)*	TiO_2-NPs of 20 nm	Single maternal IP injection	2 mg/L	Disruption of fetal brain structure; cell death via necrosis; cerebral lesions and swelling.	[22]
Mouse neonates BLAB/c strain (*Mus musculus*)	TiO_2-NPs of 35 nm	Maternal IV injection(s) (on 1 or 2 days)	100 µl/per mouse (0.8 mg per mouse)	TiO_2-NP accumulation in fetal brain; no TiO_2-NP induced cellular toxicity or DNA damage.	[23]
Adolescent female mice (*Mus musculus)*	TiO_2-NPs of 5 nm	Intranasal administration Daily 90 days	2.5, 5 and 10 mg/kg	Decreased brain weight (≥ 5 mg/kg); accumulation of TiO_2-NPs in hippocampus (≥ 5 mg/kg); alterations in hippocampal area and hippocampal neurons (≥ 2.5 mg/kg); mitochondrial swelling (2.5 mg/kg); impaired spatial recognition memory (≥ 2.5 mg/kg); reduced long term potentiation in hippocampal area (≥ 2.5 mg/kg); downregulation of NDMA receptor (subunits) (≥ 2.5 mg/kg) (all dose-dependent).	[24]

Adolescent female mice (*Mus musculus)*	TiO_2-NPs of 5 nm	Intranasal administration Daily 9 months	1.25, 2.5 and 5 mg/kg	Hippocampal edema (1.25 mg/kg and 2.5 mg/kg); hippocampal lesions (5 mg/kg); dysregulation of Glu metabolism, increased Glu release and Glu-R availability (dose-dependent) (≥ 2.5 mg/kg).	[25]
Adults female mice CD-1 strain (*Mus musculus*)	TiO_2-NPs of 5 nm	Intragastric administration Daily for 60 days	5, 10 and 50 mg/kg	Decreased brain weight (≥ 10 mg/kg); impaired spatial recognition memory (≥ 10 mg/kg); TiO_2 accumulation in brain tissue (≥ 10 mg/kg); alterations in brain structure (≥ 5 mg/kg); electrolyte content (≥ 5 mg/kg); enhanced AChE activity (≥ 5 mg/kg); decreased ATPase activity and neurotransmitter levels (NA, DA, 5-HT and metabolites) (≥ 5 mg/kg).	[26]
Adult female mice CD-1 strain (*Mus musculus*)	TiO_2-MPs of 1 µm, TiO_2-NPs of 40 nm and TiO_2-NPs with Si coating of 50 nm	Intranasal administration Once in 2 days 30 days	500 µg/mouse	Accumulation in cerebral cortex ($SiTiO_2$-NPs) and striatum ($SiTiO_2$-NPs and 40 nm TiO_2); alteration in morphology of neurons in cerebral cortex ($SiTiO_2$-NPs and 40 nm TiO_2) and hippocampus ($SiTiO_2$-NPs); decreased DA, NA and 5-HT levels (metabolites).	[27]
Adult female mice (*Mus musculus*)	TiO_2-NPs of 5 nm	Intranasal administration Daily 90 days	2.5, 5 and 10 mg/kg	Uptake and accumulation of TiO_2-NPs in brain and decreased brain weight (≥ 2.5 mg/kg); over-proliferation of glia cells (2.5 mg/kg); brain tissue necrosis (≥ 5 mg/kg); hippocampal cell death (≥ 2.5 mg/kg); oxidative stress (ROS overproduction) (≥ 2.5 mg/kg). Note, this brain injury model may have impaired barrier function.	[28]
Adult male mice (*Mus musculus)*	TiO_2-NPs of 50-75 nm	Oral exposure Daily 21 days	500 mg/kg	Accumulation in brain tissue; increased ROS in brain; increase in NA, DA and 5-HT levels in cortex region.	[29]
Wistar rat neonates (*Rattus norvegicus domestica*)	TiO_2-NPs < 30 nm (alone and co-exposed with AG)	Orally (from day 1 after fertilization until delivery, IP injection)	200 mg/kg (200 mg/kg AG)	Decreased neurogenesis, enhanced neuroinflammation (IL-6), oxidative stress (MDA and NO) and decreased antioxidant enzymes (SOD and CAT) in hippocampus, cerebellum and cerebral cortex.	[30]

Wistar rat neonates (*Rattus norvegicus domestica*)	TiO_2-NPs < 100 nm	Maternal intragastric administration Daily 20 days	100 mg/kg	Increased cell apoptosis and decreased neurogenesis in hippocampus; altered apoptosis related gene expression.	[31]
Male Wistar rat neonates (*Rattus norvegicus domestica*)	TiO_2-NPs of 10 nm	Intragastric administration; Daily from gestational day 2 to 21	100 mg/kg	Accumulation of TiO_2-NPs in hippocampus; higher brain weight/body weight ratio; decreased cell proliferation in hippocampus; impaired memory and learning.	[32]
Young adult male Wistar rats (*Rattus norvegicus domestica*)	TiO_2-NPs of 10 nm	Intratracheal instillations Daily for 28 days	1, 3 and 10 mg/kg	Increased brain weight (1 mg/kg and 3 mg/kg); slowed evoked potentials of sensory and motor neurons (3 mg/kg and 10 mg/kg): No significant particle deposition in brain.	[33]
Young adult male Wistar rats (*Rattus norvegicus domestica*)	TiO_2-NPs of 10 and 100 nm	Intratracheal instillation 5 days a week for 6 weeks	5 and 18 mg/kg	Impaired motor function (100 nm, 18 mg/kg); latency in cortical action potentials (10 ≥ 100 nm, 5 mg/kg and 18 mg/kg).	[34]
Adult male Wistar rats (*Rattus norvegicus domestica*)	TiO_2-NPs of 5-10 nm	Oral exposure Daily 60 days	50, 100 and 200 mg/kg	Decreased brain weight (≥ 100 mg/kg); decreased AChE activity plasma (≥ 50 mg/kg) and brain (≥ 100 mg/kg); increase in plasma IL-6 level (reverse dose-dependent) (≥ 50 mg/kg) and brain (dose-dependent) (≥ 100 mg/kg).	[35]
Adult male Wistar rats (*Rattus norvegicus domestica*)	TiO_2-NPs of 21 nm	IV injection single dose, 28 days post injection	5 mg/kg (10 mg/kg Ag-NPs)	Antioxidant enzyme gene expression decreased; no lipid and cholesterol peroxidation.	[36]

Adult and aged (19 months) male Fisher rats (*Rattus norvegicus domestica*)	TiO_2-NPs of 21.5 nm	Aerosol exposure, 4 weeks (5 days a week, 6 hours a day)	10 mg/m^3	Increased BBB permeability in aged rats; severe brain inflammation response in aged rats; no accumulation of particles in brain.	[37]
***In vitro* cell culture**					
Murine microglial cells (BV-2)	TiO_2-NPs of 30 nm	5, 15, 30, 45, 60 and 120 minutes 6- and 18-hours incubation	2.5, 5, 10, 20, 40, 60, 80, 100 and 120 ppm	Internalization of NPs and NP aggregation in cytoplasm (6 and 18 hours, 2.5 ppm); mitochondrial swelling (18 hours, 2.5 ppm); increased ROS (≥ 10 ppm, 30 min); no changes in cell viability.	[38]
Murine microglial cells (BV-2)	TiO_2-NPs of 20-30 nm	24 hours incubation	0.1, 1, 10, 25, 50, 100 and 200 µg/mL	Internalization of NPs (cytoplasm, vesicles and nucleus) (≥ 10 µg/mL); reduced cell viability (≥ 100 µg/mL); increased cell membrane permeability (dose-dependent) (≥ 25 µg/mL); alterations in mitochondrial transmembrane potential (≥ 200 µg/mL); ROS overproduction (≥ 10 µg/mL); no cell apoptosis.	[39]
Murine hippocampal neuron HT22 cells	TiO_2-NPs of 50 nm	6, 12, 24 and 48 hours incubation	12.5, 25, 50, 100, 200 µg/mL	Increased ROS (≥ 50 µg/mL); increased apoptosis (≥ 50 µg/m); increased apoptosis related gene expression and protein levels (≥ 100 µg/mL); down-regulation of anti-apoptosis-related proteins (≥ 200 µg/mL); reported endoplasmic reticulum stress (≥ 50 µg/mL).	[40]
Rat PC12 cells	TiO_2-NPs of 33 nm	24 hours incubation	1, 5, 25 and 125 µg/mL	Decreased cell viability (≥ 5 µg/mL); decreased DA levels (≥ 1 µg/mL); increased α-syn mRNA levels (≥ 10 µg/mL).	[41]
Rat PC12 cells	TiO_2-NPs of 20 nm (A-NP and R-NP) TiO_2-MPs 1 µm	24 hours incubation	25, 50, 100 and 200 µg/mL (A-NP) 200 µg/mL (R-NP and MP)	Decreased cell viability (A-NPs and R-NPs, dose-dependent) (A-NPs ≥ 50 µg/mL and R-NPs ≥ 100 µg/mL); impaired cell membrane integrity (A-NPs > R-NPs > MPs) (≥ 25 µg/mL); ROS generation (A-NPs > R-NPs, ≥ 100 µg/mL); increased LPO (A-NPs, ≥ 100 µg/mL); antioxidant enzyme (SOD, GSH) (A-NPs ≥ 50 µg/mL and R-NPs 200 µg/mL); mitochondrial impairment, apoptosis, necrosis and cell cycle arrest (A-NPs, ≥ 100 µg/mL > R-NPs 200 µg/mL).	[42]

Rat PC12 cells	TiO_2-NPs of 20 nm	7 days incubation	50, 100 and 200 µg/mL	Increased expression and aggregation of α-syn protein (dose-dependent) (≥ 50 µg/mL).	[43]
Rat dorsal root ganglion neurons	TiO_2-NPs of 30 nm, A/R-NPs and R-NPs	5 hours incubation	0.5 and 5 µg/mL	Internalization of NPs (0.5 and 5 µg/mL); increased apoptosis of dorsal root ganglion cells (0.5 and 5 µg/mL), sensory neurons (5 µg/mL) and glial cells (0.5 and 5 µg/mL) (dose-dependent); changes in axonal retrograde transport (5 µg/mL); ROS generation and increased inflammation (A/R-NPs only) (5 µg/mL).	[44]
Rat dorsal root ganglion neurons	TiO_2-NPs of 11-215 nm (3 A-Np types, 3 R-NP types, 4 polymorphs (80% A and 20% R)	24 hours incubation	0.5 µg/mL	Induction of apoptosis (all but uncoated rutile-NPs); cell toxicity (A/R-NPs > A-NPs >> R-NPs).	[45]
Human (U373) and rat (C6) glial cells	TiO_2-NPs < 50 nm	24 hours incubation (time-lapse microscopy)	5 µg/cm^2	TiO_2-NP internalization by both cell types via endocytosis; accumulation in cytoplasmic vesicles; internalization of TiO_2-NPs via macropinocytosis; neurotoxic effects not evaluated.	[46]
Human SH-SY5Y neuroblastoma and glial (D384) cells	TiO_2-NPs of 15 nm	4-48 hours acute<br>7-10 days prolonged incubation	1.5, 15, 31, 62, 125, 250 µg/mL (acute)<br>0.05, 0.1, 0.2, 1.5, 15, 31 µg/mL (prolonged)	Acute: Increased cell death (TiO_2-NPs ≥ bulk) in both cell lines (≥ 15 µg/mL NPs, ≥ 62 µg/mL bulk); cell membrane damage and alterations in mitochondrial functioning in glial cells and neuronal cells (≥ 125 µg/mL NPs and bulk)<br>Prolonged: TiO_2-NPs induced disruption of glial and neuronal colony formation (dose-dependent) (TiO_2-NPs only, ≥ 1.5 µg/mL).	[47]
Murine primary hippocampal neuron	TiO_2-NPs of 5 nm	24 hours incubation	5, 15 and 30 µg/mL	Reduced cell viability (dose-dependent) (≥ 5 µg/mL); internalization of NPs into the cytoplasm or nucleus (30 µg/mL); suppression of dendrite growth (dose-dependent) (≥ 5 µg/mL); increase Glu release and impaired Glu metabolism (dose-dependent) (≥ 5 µg/mL); ROS generation (≥ 15 µg/mL); decreased ATPase activity (≥ 5 µg/mL).	[48]

Human SH-SY5Y neuroblastoma cells	TiO_2-NPs of 21 nm	24 hours incubation	0.1, 1, 10 and 100 µg/mL	Alterations in cell morphology and axon retraction (100 µg/mL); accumulation of NPs in cytoplasm and lysosomes (100 µg/mL) increased ROS generation (100 µg/mL); disturbed microtubule structure (100 µg/mL); no alterations in cell viability; no accumulation in nucleus.	[49]
Human SH-SY5Y neuroblastoma cells	TiO_2-NPs of 25 nm, A-NP and A/R-NP	3, 6 (and 24 only for viability assessment) hours incubation	80, 120 and 150 µg/mL (20, 40, 60, 80, 100, 120 and 150 µg/mL viability assessment)	No decrease in cell viability and no morphological alterations; internalization of NPs (dose-dependent, A-NP > A/R-NP) (≥ 80 µg/mL); cell cycle arrest at S-phase (inverse dose-response relationship) (A-NP > A/R-NP) (≥ 80 µg/mL); increase in apoptosis (≥ 80 µg/mL); interruption of mitochondrial membrane potential (dose-dependent) (≥ 120 µg/mL, 3 hours and ≥ 80 µg/mL, 6 hours); genotoxicity (≥ 80 µg/mL); no oxidative stress.	[50]
Rat primary cortical culture	TiO_2-NPs < 100 nm	24 hours incubation	0.5, 5 and 10 µg/cm^2	Internalization of particles (TiO_2 ≥ 5 µg/cm^2); alterations in neuronal activity (1 ≥ µg/cm^2); ROS generation (TiO_2 only) (5 ≥ µg/cm^2); no alterations in neuron network morphology.	[51]
Rat primary cortical culture	TiO_2-NPs of 6, 25, 31 nm, TiO_2-MPs 0.14 µm	1, 24 and 48 hours incubation	3.1, 6.3, 12.5, 50 µg/mL	Alteration in neuronal activity (25 nm TiO_2) and number of active electrode (25 and 31 nm TiO_2).	[52]
Isolated brain mitochondria from adult male rats *in vitro*	TiO_2-NPs of 30-35 nm	1 hour incubation	5, 10 and 50 µg/mL	Significant increase in lipid peroxidation (≥ 5 µg/mL); mitochondrial protein oxidation (≥ 5 µg/mL); alteration in mitochondrial metabolism (≥ 5 µg/mL); increase in antioxidant enzyme (≥ 5 µg/mL) (all dose-dependent).	[53]

References

1. Dedeh A, Ciutat A, Treguer-Delapierre M, Bourdineaud JP. Impact of gold nanoparticles on zebrafish exposed to a spiked sediment. Nanotoxicology. 2015;9(1):71–80. https://doi.org/10.3109/17435390.2014.889238
2. Harper SL, Carriere JL, Miller JM, Hutchison JE, Maddux BLS, Tanguay RL. Systematic evaluation of nanomaterial toxicity: Utility of standardized materials and rapid assays. ACS Nano. 2011;5(6):4688–4697. https://doi.org/10.1021/nn200546k.
3. Truong L, Saili KS, Miller JM, Hutchison JE, Tanguay RL. Persistent adult zebrafish behavioral deficits results from acute embryonic exposure to gold nanoparticles. Comp Biochem Physiol C Toxicol Pharmacol 2012;155(2):269–274. https://doi.org/10.1016/j.cbpc.
4. Ferreira GK, Cardoso E, Vuolo FS, Galant LS, Michels M, Gonçalves CL, et al. Effect of acute and long-term administration of gold nanoparticles on biochemical parameters in rat brain. Materials Science and Engineering: C. 2017;79:748–755. https://doi.org/10.1016/j.msec.2017.05.110.
5. Koch F, Möller A-M, Frenz M, Pieles U, Kuehni-Boghenbor K, Mevissen M. An in vitro toxicity evaluation of gold-, PLLA- and PCL-coated silica nanoparticles in neuronal cells for nanoparticle-assisted laser-tissue soldering. Toxicology in Vitro. 2014;28(5):990–998. https://doi.org/10.1016/j.tiv.2014.04.010.
6. Trickler WJ, Lantz SM, Murdock RC, Schrand AM, Robinson BL, Newport GD, et al. Brain microvessel endothelial cells responses to gold nanoparticles: In vitro pro-inflammatory mediators and permeability. Nanotoxicology. 2010;5(4):479–492. https://doi.org/10.3109/17435390.2010.540356.
7. Trickler WJ, Lantz-Mcpeak SM, Robinson BL, Paule MG, Slikker W, Biris AS, et al. Porcine brain microvessel endothelial cells show pro-inflammatory response to the size and composition of metallic nanoparticles HHS Public Access. Drug Metab Rev. 2014;46(2):224–231. https://doi.org/10.3109/03602532.2013.873450.
8. Khalil AM. Neurotoxicity and biochemical responses in the earthworm Pheretima hawayana exposed to TiO2NPs. Ecotoxicology and Environmental Safety. 2015;122:455–461. https://doi.org/10.1016/j.ecoenv.2015.09.010.
9. Gambardella C, Aluigi MG, Ferrando S, Gallus L, Ramoino P, Gatti AM, et al. Developmental abnormalities and changes in cholinesterase activity in sea urchin embryos and larvae from sperm exposed to engineered nanoparticles. Aquatic Toxicology. 2013;130–131:77–85. https://doi.org/10.1016/j.aquatox.2012.12.025.
10. Saidani W, Sellami B, Khazri A, Mezni A, Dellali M, Joubert O, et al. (2019). Metal accumulation, biochemical and behavioral responses on the Mediterranean clams Ruditapes decussatus exposed to two photocatalyst nanocomposites (TiO2 NPs and AuTiO2NPs). Aquatic Toxicology. 2019;208:71–79. https://doi.org/10.1016/j.aquatox.2019.01.003.
11. Xia B, Zhu L, Han Q, Sun X, Chen B, Qu K. Effects of TiO2 nanoparticles at predicted environmental relevant concentration on the marine scallop Chlamys farreri: An integrated biomarker approach. Environmental Toxicology and Pharmacology. 2017;50:128–135. https://doi.org/10.1016/j.etap.2017.01.016.

12. Lüderwald S, Dackermann V, Seitz F, Adams E, Feckler A, Schilde C, et al. A blessing in disguise? Natural organic matter reduces the UV light-induced toxicity of nanoparticulate titanium dioxide. Science of the Total Environment. 2019;663:518–526. https://doi.org/10.1016/j.scitotenv.2019.01.282.
13. Hu Q, Guo F, Zhao F, Fu Z. Effects of titanium dioxide nanoparticles exposure on parkinsonism in zebrafish larvae and PC12. Chemosphere. 2017;173:373–379. https://doi.org/10.1016/j.chemosphere.2017.01.063.
14. Wang YJ, He ZZ, Fang YW, Xu Y, Chen YN, Wang GQ, et al. Effect of titanium dioxide nanoparticles on zebrafish embryos and developing retina. International Journal of Ophthalmology. 2014;7(6): 917–923. https://doi.org/10.3980/j.issn.2222-3959.2014.06.01.
15. Li M, Wu Q, Wang Q, Xiang D, Zhu G. Effect of titanium dioxide nanoparticles on the bioavailability and neurotoxicity of cypermethrin in zebrafish larvae. Aquatic Toxicology. 2018;199:212–219. https://doi.org/10.1016/j.aquatox.2018.03.022.
16. Miao W, Zhu B, Xiao X, Li Y, Dirbaba NB, Zhou B, Wu H. Effects of titanium dioxide nanoparticles on lead bioconcentration and toxicity on thyroid endocrine system and neuronal development in zebrafish larvae. Aquatic Toxicology. 2015;161:117–126. https://doi.org/10.1016/j.aquatox.2015.02.002.
17. Wang Q, Chen Q, Zhou P, Li W, Wang J, Huang C, et al. Bioconcentration and metabolism of BDE-209 in the presence of titanium dioxide nanoparticles and impact on the thyroid endocrine system and neuronal development in zebrafish larvae. Nanotoxicology. 2014;8(SUPPL. 1):196–207. https://doi.org/10.3109/17435390.2013.875232.
18. Sheng L, Wang L, Su M, Zhao X, Hu R, Yu X, et al. Mechanism of TiO2 nanoparticle-induced neurotoxicity in zebrafish (Danio rerio). Environmental Toxicology. 2016;31(2):163–175. https://doi.org/10.1002/tox.22031.
19. Carmo TLL, Siqueira PR, Azevedo VC, Tavares D, Pesenti EC, Cestari MM, et al. Overview of the toxic effects of titanium dioxide nanoparticles in blood, liver, muscles, and brain of a Neotropical detritivorous fish. Environmental Toxicology. 2019;34(4):457–468. https://doi.org/10.1002/tox.22699.
20. Miranda RR, Damaso da Silveira ALR, de Jesus IP, Grötzner SR, Voigt CL, Campos SX, et al. Effects of realistic concentrations of TiO 2 and ZnO nanoparticles in Prochilodus lineatus juvenile fish. Environmental Science and Pollution Research. 2016;23(6):5179–5188. https://doi.org/10.1007/s11356-015-5732-8.
21. Hong F, Zhou Y, Ji J, Zhuang J, Sheng L, Wang L. Nano-TiO2 inhibits development of the central nervous system and its mechanism in offspring mice. Journal of Agricultural and Food Chemistry. 2018;66(44):11767–11774. https://doi.org/10.1021/acs.jafc.8b02952.
22. Naserzadeh P, Ghanbary F, Ashtari P, Seydi E, Ashtari K, Akbari M. Biocompatibility assessment of titanium dioxide nanoparticles in mice fetoplacental unit. Journal of Biomedical Materials Research - Part A. 2018;106(2):580–589. https://doi.org/10.1002/jbm.a.36221.
23. Yamashita K, Yoshioka Y, Higashisaka K, Mimura K, Morishita Y, Nozaki M, et al. Silica and titanium dioxide nanoparticles cause pregnancy complications in mice. Nature Nanotechnology. 2011;6(5):321–328. https://doi.org/10.1038/nnano.2011.41.

24. Ze Y, Sheng L, Zhao X, Ze X, Wang X, Zhou Q, et al. Neurotoxic characteristics of spatial recognition damage of the hippocampus in mice following subchronic peroral exposure to TiO2 nanoparticles. Journal of Hazardous Materials 2013;264:219–229. https://doi.org/10.1016/j.jhazmat.2013.10.072.
25. Ze X, Su M, Zhao X, Jiang H, Hong J, Yu X, et al. TiO2nanoparticle-induced neurotoxicity may be involved in dysfunction of glutamate metabolism and its receptor expression in mice. Environmental Toxicology. 2014;31(6):655–662. https://doi.org/10.1002/tox.22077.
26. Hu R, Gong X, Duan Y, Li N, Che Y, Cui Y, et al. Neurotoxicological effects and the impairment of spatial recognition memory in mice caused by exposure to TiO2 nanoparticles. Biomaterials. 2010;31(31):8043–8050. https://doi.org/10.1016/j.biomaterials.2010.07.011.
27. Zhang L, Bai R, Li B, Ge C, Du J, Liu Y, et al. Rutile TiO2 particles exert size and surface coating dependent retention and lesions on the murine brain. Toxicology Letters. 2011;207(1):73–81. https://doi.org/10.1016/j.toxlet.2011.08.001.
28. Ze Y, Hu R, Wang X, Sang X, Ze X, Li B, et al. Neurotoxicity and gene-expressed profile in brain-injured mice caused by exposure to titanium dioxide nanoparticles. Journal of Biomedical Materials Research - Part A. 2013;102(2):470–478. https://doi.org/10.1002/jbm.a.34705.
29. Shrivastava R, Raza S, Yadav A, Kushwaha P, Flora SJS. Effects of sub-acute exposure to TiO2, ZnO and Al2O3 nanoparticles on oxidative stress and histological changes in mouse liver and brain. Drug and Chemical Toxicology. 2014;37(3):336–347. https://doi.org/10.3109/01480545.2013.866134.
30. Asghari A, Hosseini M, Beheshti F, Shafei MN, Mehri S. Inducible nitric oxide inhibitor aminoguanidine, ameliorated oxidative stress, interleukin-6 concentration and improved brain-derived neurotrophic factor in the brain tissues of neonates born from titanium dioxide nanoparticles exposed rats. Journal of Maternal-Fetal and Neonatal Medicine. 2019:32(23):3962–3973. https://doi.org/10.1080/14767058.2018.1480602.
31. Bideskan AE, Mohammadipour A, Fazel A, Haghir H, Rafatpanah H, Hosseini M, Rajabzadeh A. Maternal exposure to titanium dioxide nanoparticles during pregnancy and lactation alters offspring hippocampal mRNA BAX and Bcl-2 levels, induces apoptosis and decreases neurogenesis. Experimental and Toxicologic Pathology. 2017;69(6):329–337. https://doi.org/10.1016/j.etp.2017.02.006.
32. Mohammadipour A, Fazel, A, Haghir H, Motejaded F, Rafatpanah H, Zabihi H, et al. Maternal exposure to titanium dioxide nanoparticles during pregnancy; impaired memory and decreased hippocampal cell proliferation in rat offspring. Environmental Toxicology and Pharmacology. 2014;37(2):617–625. https://doi.org/10.1016/j.etap.2014.01.014.
33. Horváth T, Papp A, Kovács D, Kálomista I, Kozma G, Vezér T. Electrophysiological alterations and general toxic signs obtained by subacute administration of titanium dioxide nanoparticles to the airways of rats. Ideggyogy Sz. 2017;70(3-4):137–135. https://doi.org/10.18071/isz.70.0127.
34. Horváth T, Vezér T, Kozma G, Papp A. Functional neurotoxicity and tissue metal levels in rats exposed subacutely to titanium dioxide Nanoparticles via the airways. Ideggyogy Sz. 2018;71(1–2):35–42. https://doi.org/10.18071/isz.71.0035.

35. Grissa I, Guezguez S, Ezzi L, Chakroun S, Sallem A, Kerkeni E, et al. The effect of titanium dioxide nanoparticles on neuroinflammation response in rat brain. Environmental Science and Pollution Research. 2016;23(20):20205–20213. https://doi.org/10.1007/s11356-016-7234-8.
36. Krawczyńska A, Dziendzikowska K, Gromadzka-Ostrowska J, Lankoff A, Herman AP, Oczkowski M, et al. Silver and titanium dioxide nanoparticles alter oxidative/inflammatory response and renin–angiotensin system in brain. Food and Chemical Toxicology. 2015;85:96–105. https://doi.org/10.1016/j.fct.2015.08.005.
37. Disdier C, Chalansonnet M, Gagnaire F, Gaté L, Cosnier F, Devoy J, et al. Brain inflammation, blood brain barrier dysfunction and neuronal synaptophysin decrease after inhalation exposure to titanium dioxide nano-aerosol in aging rats. Scientific Reports. 2017;7(1):12196. https://doi.org/10.1038/s41598-017-12404-5.
38. Long TC, Saleh N, Tilton RD, Lowry GV, Veronesi B. Titanium dioxide (P25) produces reactive oxygen species in immortalized brain microglia (BV2): Implications for nanoparticle neurotoxicity. Environmental Science and Technology. 2006;40(14):4346–4352. https://doi.org/10.1021/es060589n.
39. Rihane N, Nury T, M'rad I, El Mir L, Sakly M, Amara S, Lizard G. Microglial cells (BV-2) internalize titanium dioxide (TiO2) nanoparticles: toxicity and cellular responses. Environmental Science and Pollution Research. 2016;23(10):9690–9699. https://doi.org/10.1007/s11356-016-6190-7.
40. He Q, Zhou X, Liu Y, Gou W, Cui J, Li Z, et al. Titanium dioxide nanoparticles induce mouse hippocampal neuron apoptosis via oxidative stress- and calcium imbalance-mediated endoplasmic reticulum stress. Environmental Toxicology and Pharmacology. 2018;63:6–15. https://doi.org/10.1016/j.etap.2018.08.003.
42. Wu J, Sun J, Xue Y. Involvement of JNK and P53 activation in G2/M cell cycle arrest and apoptosis induced by titanium dioxide nanoparticles in neuron cells. Toxicology Letters. 2010:199(3):269–276. https://doi.org/10.1016/j.toxlet.2010.09.009.
43. Wu J, Xie H. Effects of titanium dioxide nanoparticles on a-synuclein aggregation and the ubiquitin-proteasome system in dopaminergic neurons. Artificial Cells, Nanomedicine and Biotechnology. 2016;44(2):690–694. https://doi.org/10.3109/21691401.2014.980507.
44. Erriquez J, Bolis V, Morel S, Fenoglio I, Fubini B, Quagliotto P, Distasi C. Nanosized TiO2 is internalized by dorsal root ganglion cells and causes damage via apoptosis. Nanomedicine: Nanotechnology, Biology and Medicine. 2015;11(6):1309–1319. https://doi.org/10.1016/j.nano.2015.04.003.
45. Bolis V, Busco C, Ciarletta M, Distasi C, Erriquez J, Fenoglio I, et al. Hydrophilic/hydrophobic features of TiO2 nanoparticles as a function of crystal phase, surface area and coating, in relation to their potential toxicity in peripheral nervous system. Journal of Colloid and Interface Science. 2012;369(1):28–39. https://doi.org/10.1016/j.jcis.2011.11.058.
46. Huerta-García E, Márquez-Ramírez SG, Ramos-Godinez M. del P, López-Saavedra A, Herrera LA, Parra A, et al. Internalization of titanium dioxide nanoparticles by glial cells is given at short times and is mainly mediated by actin reorganization-dependent endocytosis. NeuroToxicology. 2015;51:27–37. https://doi.org/10.1016/j.neuro.2015.08.013.

47. Coccini T, Grandi S, Lonati D, Locatelli C, De Simone U. Comparative cellular toxicity of titanium dioxide nanoparticles on human astrocyte and neuronal cells after acute and prolonged exposure. NeuroToxicology. 2015;48:77–89. https://doi.org/10.1016/j.neuro.2015.03.006.
48. Hong F, Sheng L, Ze Y, Hong J, Zhou Y, Wang L, et al. Suppression of neurite outgrowth of primary cultured hippocampal neurons is involved in impairment of glutamate metabolism and NMDA receptor function caused by nanoparticulate TiO2. Biomaterials. 2015;53:76–85. https://doi.org/10.1016/j.biomaterials.2015.02.067.
49. Mao Z, Xu B, Ji X, Zhou K, Zhang X, Chen M, et al. Titanium dioxide nanoparticles alter cellular morphology via disturbing the microtubule dynamics. Nanoscale. 2015;7(18):8466–8475. https://doi.org/10.1039/c5nr01448d.
50. Valdiglesias V, Costa C, Sharma V, Kiliç G, Pásaro E, Teixeira JP, et al. Comparative study on effects of two different types of titanium dioxide nanoparticles on human neuronal cells. Food and Chemical Toxicology. 2013;57:352–361. https://doi.org/10.1016/j.fct.2013.04.010.
51. Gramowski A, Flossdorf J, Bhattacharya K, Jonas L, Lantow M, Rahman Q, et al. Nanoparticles induce changes of the electrical activity of neuronal networks on microelectrode array neurochips. Environmental Health Perspectives. 2010;118(10):1363–1369. https://doi.org/10.1289/ehp.0901661.
52. Strickland JD, Lefew WR, Crooks J, Hall D, Ortenzio JN, Dreher K, Shafer TJ. In vitro screening of metal oxide nanoparticles for effects on neural function using cortical networks on microelectrode arrays. Nanotoxicology. 2016;10(5):619–628. https://doi.org/10.3109/17435390.2015.1107142.
53. Nalika N, Parvez S. Mitochondrial dysfunction in titanium dioxide nanoparticle-induced neurotoxicity. Toxicology Mechanisms and Methods. 2015;25(5):355–363. https://doi.org/10.3109/15376516.2015.1020183.
